# Pre-metastatic niche: formation, characteristics and therapeutic implication

**DOI:** 10.1038/s41392-024-01937-7

**Published:** 2024-09-25

**Authors:** Yuhang Wang, Jiachi Jia, Fuqi Wang, Yingshuai Fang, Yabing Yang, Quanbo Zhou, Weitang Yuan, Xiaoming Gu, Junhong Hu, Shuaixi Yang

**Affiliations:** 1 Department of Colorectal Surgery, The First Affiliated Hospital of Zhengzhou University, 1 East Jianshe Road, Zhengzhou, 450000 China; 2 College of Medicine, Zhengzhou University, Zhengzhou, 450001 China

**Keywords:** Cancer microenvironment, Immunotherapy, Cancer therapy

## Abstract

Distant metastasis is a primary cause of mortality and contributes to poor surgical outcomes in cancer patients. Before the development of organ-specific metastasis, the formation of a pre-metastatic niche is pivotal in promoting the spread of cancer cells. This review delves into the intricate landscape of the pre-metastatic niche, focusing on the roles of tumor-derived secreted factors, extracellular vesicles, and circulating tumor cells in shaping the metastatic niche. The discussion encompasses cellular elements such as macrophages, neutrophils, bone marrow-derived suppressive cells, and T/B cells, in addition to molecular factors like secreted substances from tumors and extracellular vesicles, within the framework of pre-metastatic niche formation. Insights into the temporal mechanisms of pre-metastatic niche formation such as epithelial-mesenchymal transition, immunosuppression, extracellular matrix remodeling, metabolic reprogramming, vascular permeability and angiogenesis are provided. Furthermore, the landscape of pre-metastatic niche in different metastatic organs like lymph nodes, lungs, liver, brain, and bones is elucidated. Therapeutic approaches targeting the cellular and molecular components of pre-metastatic niche, as well as interventions targeting signaling pathways such as the TGF-β, VEGF, and MET pathways, are highlighted. This review aims to enhance our understanding of pre-metastatic niche dynamics and provide insights for developing effective therapeutic strategies to combat tumor metastasis.

## Introduction

Cancer metastasis, which involves the dissemination of cancer cells from a primary lesion to distal organs, is the leading cause of cancer-related death.^1,2^ Metastasis occurs as genetically unstable cancer cells adapt to a tissue microenvironment distant from the primary tumor site.^3,4^ For decades, research into cancer metastasis has primarily concentrated on the causes of oncogenic transformation and the initial onset of tumor development.^5^ Tumor metastasis is usually associated with a poor prognosis.^6,7^ Although many therapies have been developed for cancer treatment, metastasis continues to be a significant contributor to cancer-related deaths.

Metastasis of tumor cells has already been described as a specific process in which a great quantity of tumor cells leave their primary site, circulate in peripheral blood, pass through blood vessels and finally settle into a distant organ.^8^ With new discoveries about the tumor microenvironment (TME), cancer metastasis into particular sites can be explained as a process in which a specific microenvironment plays a key role in trapping tumor cells.^9^ This is the moment when the notion of pre-metastatic niche (PMN) is proposed, which specifically refers to the circumstances at future metastatic sites.^10^ The PMN represents a complex microenvironment, crafted through the intricate interplay of numerous bone marrow-derived cells (BMDCs) and various molecular constituents. Both cellular constituents and molecular elements collaborate to reshape the microenvironment, priming distant organs for the metastasis of tumor cells. Following the establishment of PMN, the microenvironment is marked by immunosuppression, enhanced vascular permeability, and angiogenesis, all of which are all vital for the settlement and proliferation of tumor cells.^11^

**Fig. 1:**
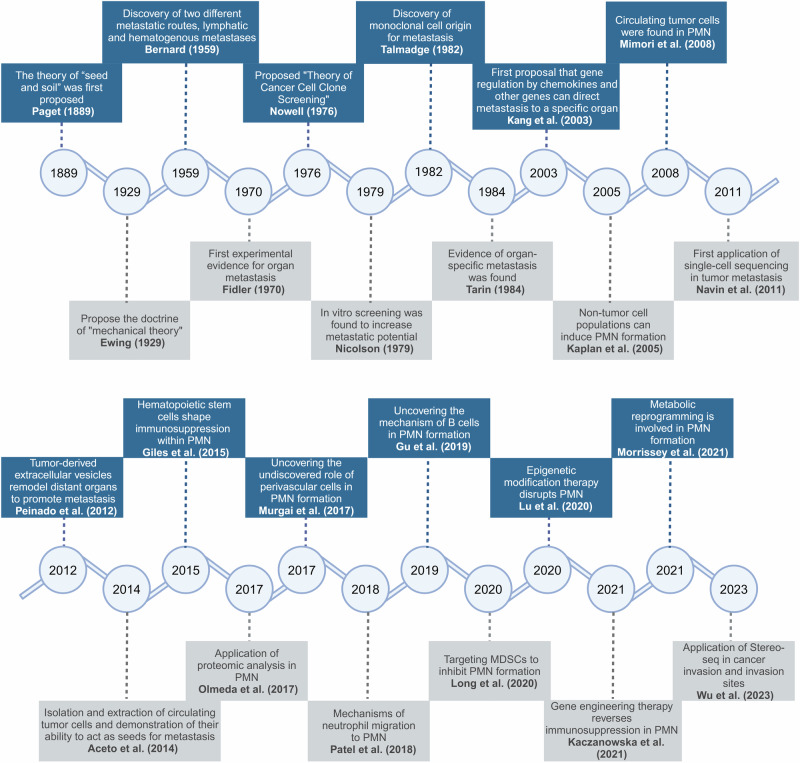
Historical progression in pre-metastatic niche research: Unraveling the journey to understanding. From initial discoveries to current advancements, researchers have unraveled the complexities of the pre-metastatic niche, shedding light on the mechanisms involved in preparing secondary sites for tumor metastasis. This journey of exploration and discovery has led to significant insights that may ultimately inform novel therapeutic strategies for preventing or treating metastatic disease

More than a century ago, Paget first posited the “seed and soil hypothesis”, and since then, researchers have been exploring the mechanisms of cancer metastasis.^12^ Subsequently, Lyden was the first to introduce the concept of the PMN.^10^ Increasing evidence in recent literature indicates that tumor establish the PMN in the target organ before metastasis occurs, providing metastatic tumor cells with a microenvironment suitable and supportive of their colonization.^11,13–15^ The PMN has been studied for quite a long time from its discovery to the mechanisms revealed today (Fig. 1). The PMN is characterized as a conducive and hospitable tissue microenvironment that undergoes diverse molecular and cellular alterations to establish locations earmarked for metastasis, or a fertile “soil” prepared for the colonization of metastatic tumor cell “seed”, thereby facilitating tumor settlement in distant organs and promoting tumor metastasis.^11,16^ There is increasing recognition of the function and importance of the PMN in the process of metastasis.^17,18^

Typically, the formation of the PMN involves three primary factors: primary tumor-derived constituents, tumor-mobilized BMDCs and the local stromal microenvironment of prospective metastatic organs.^5,19,20^ It is nearly certain that preventing the establishment of PMN can effectively reduce cancer mortality and improve the effectiveness of immunotherapy.^19,21^ This review comprehensively elucidates the molecular mechanisms underlying the PMN, with a specific focus on its formation and characteristics. Furthermore, we anticipate future advancements in cancer treatment.

## Pre-metastatic Niche provides soil for distant metastasis of cancer

**Fig. 2:**
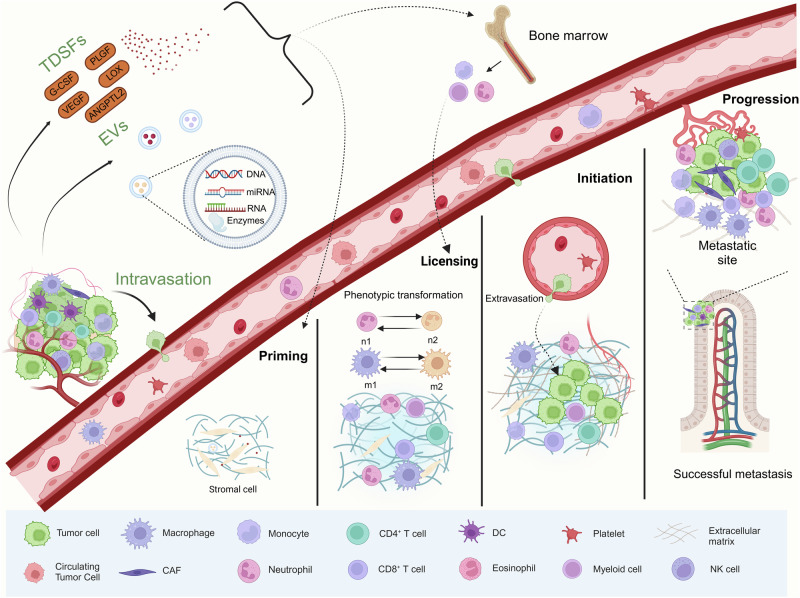
Tracing the path from primary tumor to pre-metastatic niche formation: Unraveling the sequential events in metastasis development. This description outlines the evolving tumor microenvironment at various stages of cancer progression, and demonstrates key representative cell types. The primary tumor promotes the formation of the pre-metastatic niche through the release of various cells and factors. Circulating tumor cells entering the pre-metastatic niche form the tumor microenvironment of distant metastasis

The influence of developing tumors on the host extends beyond the local TME.^22,23^ Notably, primary tumors can prompt the establishment of microenvironments in distant organs that are conducive to cancer cell growth through paracrine effects, a phenomenon known as the PMN.^14,24^ The establishment of PMN involves a complex assortment of cellular components and molecular components.^25^ It has been demonstrated that these cellular components and molecular components undergo transformation in response to signals from the primary tumor, leading to the conversion of originally healthy and resistant secondary organs and tissues into a supportive “soil” for the colonization of metastatic tumor cells.^21^ At the same time, we show the detailed process from the primary tumor stage to the establishment of pre-metastatic microenvironment and the subsequent development of metastatic foci (Fig. 2).

### Involved cellular components in pre-metastatic niche formation

#### Macrophages

Macrophages are a critical component of the TME, comprising over half of the total of tumor cell population.^26^ Mounting evidence emphasizes the pivotal role of macrophages in the establishment of metastatic niches.^27–31^ The involvement of macrophages in the development of PMN is multifaceted. Macrophages, which are terminally differentiated cells derived from monocytes in the mononuclear phagocytic system, are abundant throughout tumor progression.^32^ Macrophages residing within tumors are referred to as tumor-associated macrophages (TAMs), comprising both M1 and M2 phenotypes.^33^ M1 macrophages exhibit antitumorigenic properties, whereas M2 macrophages promote tumor growth.^34^ In contrast, macrophages present at sites of tumor metastasis include not only bone marrow-derived macrophages (BMDMs) but also tissue-resident macrophages (TRMs) such as Kupffer cells and subcapsular sinus macrophages.^35–37^ During the establishment of PMN, the phenotype of macrophages undergoes changes in response to secreted factors from tumor cells and stromal cells, which contributes to different roles in metastasis.

In the early phases of PMN formation, macrophages and TRMs within the pre-existing microenvironment of the distal organ are capable of antigen presentation and immune response.^38^ Then, uncontrolled proliferation, hypoxia and inflammation occur at the primary tumor site. These processes lead to the generation of diverse tumor-derived secreted factors (TDSFs), extracellular vesicles (EVs) and other molecular components.^11^ These molecular components are released into the blood circulation to metastasize to future metastatic organs and recruit inhibitory or regulatory immune cells, including macrophages.^39,40^ Immune cells such as macrophages recruited into the pre-metastatic ecotone first create an inflammatory environment, which induces more macrophages to accumulate. This leads to the formation of a vicious cycle locally in the PMN, where signals like exosomes and soluble factors released by the primary tumor further additionally stimulate the conversion of macrophages toward the M2 phenotype. A recent study indicates that caveolin-1 (Cav-1) present in exosomes originating from breast cancer cells can promote the establishment of PMN by inducing the upregulation of genes linked to PMN formation in lung epithelial cells and facilitating the M2 polarization of lung macrophages.^41^ In addition, Cav-1-containing exosomes inhibit the PTEN/CCL2/VEGF-A signaling pathway and promote pulmonary angiogenesis as well as M2 polarization of lung macrophages.^42^

As TDSFs, BMDCs, and EVs accumulate at the metastatic site, the microenvironment gradually transformed into PMN suitable for the colonization and growth of tumor cells. Macrophages in the pre-metastatic microenvironment are recruited, and TRMs influenced by the primary tumor also play a role in the development of metastasis.^43^ Studies have shown that osteoblasts, as TRMs, can promote bone development.^44^ Breast tumor cells are able to secrete LOX, which promotes osteoclastogenesis by activating the NFATc1 transcription factor. Excessive osteoclasts promote bone resorption, ultimately facilitating the formation of PMN.^45^ These macrophages interact with soluble mediators derived from primary tumors and gradually transform into phenotypes that promote immunosuppression and angiogenesis.^46,47^ TAMs facilitate the recruitment of Treg cells to the PMN through the secretion of CCL22.^48^ Macrophages display functional plasticity in response to local microenvironmental cues and contribute to cancer-related inflammation, extracellular matrix (ECM) remodeling, immune evasion, and ultimately cancer metastasis. Tumor-derived factors stimulate resident macrophages at pre-metastatic locations, prompting the recruitment of CD11b + Ly6c-high inflammatory monocytes that gather and evolve into metastasis-associated macrophages.^49^ Metastasis-associated macrophages assist in circulating tumor cells (CTCs) extravasation, migration through the matrix and the formation of micrometastases.^50,51^ With the maturation of PMN, CTCs are promoted to infiltrate from blood vessels and attract tumor cells to the niche to actively promote the occurrence of metastasis. Finally, as micrometastasis develop into significant large metastasis, the macrophage phenotype is transformed into TAM. These TAMs inhibit the immune response, promote angiogenesis, stimulate tumor cell infiltration and metastasis, and support tumor growth at metastatic sites.^52–54^ In summary, macrophages assume a pivotal role in oncogenesis. They not only contribute to tumor promotion at primary sites, but also facilitate the formation of PMNs conducive to CTC colonization at metastatic sites.^55^ Overall, the polarization, expansion and recruitment of macrophages are crucial in the establishment of the PMN.

#### Neutrophils

Neutrophils are pivotal both in terms of quantity and function at distant metastatic sites. The factors that recruit neutrophils from circulating blood can be primarily classified into three major categories- chemotactic factors, EVs and other bioactive factors.^56^ The most well-elucidated chemotactic factors are members of the C-X-C motif chemokine ligand (CXCL) family. CXCL8(IL-8)/CXCR2 is probably the most common chemotactic axis that they are found to be abundantly functioned in the distal sites and IL-8 is discharged by tumor cells and stromal cells across diverse malignancies, including breast cancer, colorectal cancer, cervical cancer, and acute myeloid leukemia.^57^ And it is noteworthy that in Yu’s study focusing on murine models with deficient CXCR2 expression, fewer neutrophil infiltrations and occurrences of metastasis in distant organs have been observed.^58^ EVs represent single-membrane vesicles pivotal in facilitating long-range intercellular communication through their cargo, including DNA, RNA and certain proteins. Tumor-derived EVs, in particular, have emerged as critical components in the orchestration of the premetastatic niche. In a recent investigation, toll-like receptor 3 (TLR3) expressed on lung epithelial cells was identified as the catalyst instigating neutrophil recruitment and the formation of a PMN in the lung. This occurs through the detection of tumor-derived exosomal RNA by TLR3, thereby stimulating the secretion of CXCL5 and CXCL12.^59^ Prominent representatives of bioactive molecules include myeloid-related proteins such as S100A8 and S100A9, which are abundantly expressed at metastatic sites and serve as potent chemoattractants for neutrophils.^60^ However, the exact mechanism driving MRPs-mediated neutrophil mobilization remains elusive to date and more in-depth explorations are warranted. Another crucial bioactive molecule is the downstream product of hypoxia inducible factor-1 - vascular endothelial growth factor (VEGF) which can recruit neutrophils and mediate their adhesion to postcapillary venules.^61^

Upon attraction to distant metastatic organs, neutrophils undergo a series of phenotypic alterations influenced by the surrounding microenvironment to facilitate PMN formation. Neutrophils in cancer exhibit heterogeneity, manifesting both pro-tumorigenic phenotype (N1) and anti-tumorigenic phenotype (N2).^62^ The complex microenvironment engaged in PMN formation greatly affects neutrophil differentiation.^63^ Type I interferons (IFNs) are pivotal in modulating immune responsiveness by guiding the polarization of neutrophils toward the N1 phenotype.^64^ Recent investigations concerning murine models, it has been observed that the lack of type I IFNs often not only lead to significant aggregation of neutrophils and their transition towards the N2 phenotype but also leads to the secretion of substantial amounts of neutrophil-derived immunosuppressive molecules such as S100A8, S100A9, and Bv8.^60,65,66^ Not only does the reduction of IFNs contribute to the establishment of the PMN, but the secretion of IL-6, IL-10, and granulocyte colony-stimulating factor (G-CSF) by tumor cells also induces the transition of neutrophils towards the N2 phenotype upon arrival via bloodstream at pre-metastatic sites to create an immunosuppressive environment.^59^ However, this does not imply that all neutrophils in the PMN population will convert to the N2 phenotype. In most cases, neutrophils in PMN predominantly exhibit in a coexisting N1 and N2 phenotypes.

Metabolic alterations are another important change made by neutrophils to facilitate the successful metastasis and growth of tumors. Metabolic changes involve the upregulation of fatty acid transport protein 2 (FATP2) and elevated levels of Arg1, both of which inhibit the tumoricidal activity of CD8 + T cells to some extent.^67–69^ The heightened expression of FATP2 leads to neutrophils avidly engulfing lipids and metabolizing them into prostaglandin E2 (PGE2) which could cause T cell suppression via the PGE2-EP2/EP4 signaling pathway.^70^ ARG1 impedes T cell proliferation and functionality by depleting the indispensable amino acid L-arginine, which is crucial for T cell activation and proliferation as well as promoting the polarization of macrophages toward an immunosuppressive M2-like phenotype.^68^ Another altered metabolic pathway is glucose metabolism, and studies have already found that tumor-associated neutrophils exhibit elevated rates of glycolysis and oxidative phosphorylation compared to those of neutrophils in normal tissues.^71^ Neutrophil extracellular traps (NETs), a newly discovered structure derived from activated neutrophils, have recently been shown to reshape the ECM, awaken dormant cancer cells and metastasize them.^72^ In the future, elucidating the precise mechanisms underlying accelerated glycolysis will remain imperative.

#### Myeloid-derived suppressor cells

Myeloid-derived suppressor cells (MDSCs), which are mainly derived from hematopoietic stem cells, constitute a cohort of immature myeloid cells.^73^ Under certain physiological conditions, these cells are capable of migrating to peripheral organs and differentiating into dendritic cells (DCs), macrophages, or granulocytes.^74,75^ They possess potent immunosuppressive capabilities, accumulate in cancer patients, and are critical in the early formation of PMN.^76^ Under typical physiological circumstances, MDSCs are predominantly located in the bone marrow and spleen, while tumor-secreted signaling molecules facilitate the recruitment of MDSCs to target organs.^77,78^ Some scholars have summarized the phenotype, morphology and function of MDSCs, and divided them into polymorphonuclear-MDSCs, early-MDSCs and monocytic-MDSCs.^79^ Related studies have shown that the majority of MDSCs in mice exhibit the granulocyte phenotype CD11b^+^ Ly6C^lo^ Ly6G^+^.^80^ However, hypoxia in the PMN can trigger MDSCs to transform into nonspecific inhibitory cells and differentiate into macrophages that produce high levels of IL-10, ARG1, iNOS, IL-12, and IL-6.^81,82^

Studies have shown that tumor cells at the primary site are capable of secreting VEGF-A, which in turn stimulates the production of CXCL1 by TAMs at the primary site. This leads to the recruitment of CXCR2-positive MDSCs through the CXCL1-CXCR2 signaling pathway in the liver, ultimately resulting in the establishment of a PMN. The buildup of MDSCs in the pre-metastatic microenvironment promotes the survival of CTCs, thus facilitating the development of liver metastases in colorectal cancer.^83^ Interestingly, chronic psychological stress can stimulate the release of glucocorticoids in the body, leading to the induction of CXCL1 secretion by TAMs. Consequently, this process enhances the proliferation, migration, and suppression of CD8 + T cells by MDSCs via CXCR2, thereby crucially contributing to the establishment of the PMN.^84^ Consequently, the accumulation of MDSCs promotes the formation of PMN and liver metastasis. Tumor-derived chemokines such as granulocyte-macrophage colony stimulating factor (GM-CSF) and PGE2 recruit MDSCs to remote secondary sites.^85^ These findings highlight the diverse mechanisms through which MDSCs play a pivotal role in the formation of the PMN, underscoring their essential contribution to the successful establishment of this critical environment.

In previous studies, the fundamental mechanisms through which MDSCs impede the host immune response by eliminating tumor cells were explored: (a) Generating immunosuppressive factors such as transforming growth factor β (TGF-β) and reactive oxygen species (ROS) which can suppress T cell cytotoxicity.^86^ (b) The accumulation of MDSCs in PMN may induce natural killer cell (NK cell) anergy, thereby diminishing the cytotoxicity against tumor cells.^87^ (c) MDSCs can induce the recruitment of Treg cells, which are known for their immunosuppressive effects.^85^ Furthermore, MDSCs can facilitate the formation of the pre-metastatic microenvironment by inhibiting immune cells and secreting many proangiogenic factors to promote angiogenesis.^88,89^ In conclusion, MDSCs play a crucial role in the formation of the PMN. More in-depth studies have shown that MDSCs can not only inhibit cancer metastasis, but also effectively reduce the recurrence rate of cancer.^90^

#### T and B cells

T and B cells play crucial roles in cancer by engaging in immune responses through killing cancer cells, regulating immune responses, and producing antibodies, thereby assisting the body in combating the initiation and dissemination of cancer.^91,92^ Conversely, under particular circumstances, such as regulatory B cells and regulatory T cells (Tregs), T and B cells can also act in an immunosuppressive capacity.^93^ They are also essential for the establishment of PMN. Generally, they function as the regulated, being countered by other cells or molecular components before CTCs reach the metastatic site, thus contributing to the immunosuppressive microenvironment of cancer.^94^

Throughout the establishment of PMN, the reduction of cytotoxic and effector T cells, along with the rise in Tregs, plays a crucial role in creating T cell-mediated immunosuppression. Similarly, alveolar macrophages can accumulate in the metastasis niche via complement C5a receptor-mediated proliferation. They subsequently regulate TGF-β within the lung environment, diminishing both the quantity and maturation of lung DCs. This process weakens the Th1 response while enhancing the Th2 response, thereby diminishing the efficacy of anti-tumor T cells.^95^ In the PMN, IL-6 and IL-10 can induce neutrophil N2 conversion, resulting in a decrease in IL-12a levels.^96^ IL-12a is a key factor in Th1 differentiation, and its reduction leads to weakened Th1 differentiation of CD4 + T cells, while a lack of Th1 subset leads to insufficient activation and immunosuppression of CD8+ CTLs.^97,98^ The infiltration of CD8 + T cells is inversely proportional to the activity of STAT3.^99^ Decreasing STAT3 levels in myeloid cells within the pre-metastatic microenvironment leads to aberrant activation of CD8 + T cells. This directly inhibits the function of myeloid cells, ultimately facilitating the colonization of CTCs in the PMN.^100^ Recent research indicates that T cells and B cells can synergistically facilitate the formation of pre-metastatic bone tissue in breast cancer at specific concentrations.^101^

In addition to the internal factors of the body, chemotherapy also induces the formation of premetastatic niches.^102,103^ In a related study, patients receiving oxaliplatin showed a wide range of immunosuppressive macrophages and non-reactive T cells in the liver, which facilitated the colonization of CTCs.^104^ In addition, surgical trauma and stress can also regulate the body’s T cell function leading to immunosuppression.^105^ Studies have demonstrated that following surgery, there is an increase in the proliferation and activation of Tregs. This leads to the suppression of anti-tumor effector cells such as cytotoxic T cells and NK cells, contributing to immunosuppression and aiding in the establishment of the PMN.^106^

B cells mainly secrete antibodies to participate in humoral immunity. In previous studies, mice with breast cancer were able to produce IgG that targets the glycosylated membrane protein HSPA4, thereby activating the CXCR4/SDF1α axis to promote lymph node metastasis. However, there remains a lack of clear evidence illuminating the function of B cells in the development of the PMN, underscoring the imperative for more comprehensive investigation.^9^

### Involved molecular components in pre-metastatic niche formation

#### Tumor-derived secreted factors

Tumors, as complex diseases, not only invade and damage surrounding tissues, but also secrete a large number of signaling molecules (TDSFs) under conditions of inflammation and hypoxia to engage in intercellular communication with other tissues and cells in the body.^107^ TDSFs are soluble proteins secreted by tumor cells that facilitate the establishment of PMN through various mechanisms.^11,108,109^ Table 1 provides a list of molecular components released by primary tumors that promote the formation of the PMN. Numerous studies have demonstrated that TDSFs can directly mobilize and recruit myeloid cells from the bone marrow into the PMN, promoting its formation.^59,110^

**Table 1:** Molecular components released by primary tumors that promote the formation of Pre-metastatic Niche

Tumor secretes soluble mediators	Molecules	Function and mechanism	Tumor metastasis models	Refs.
Tumor-derived secreted factors	EGF	EGF mediates cancer metastasis by regulating SNX3 protein expression and interaction with EGFR	Breast cancer	^74^
	FGF	FGF-2 induces CXCL14 expression via the FGFR1/AHR signaling pathway, leading to enhanced macrophage recruitment and polarization towards an M2-like phenotype	Nasopharyngeal carcinoma	^76^
	VEGF	Recruit VEGFR1+ bone marrow-derived HPCs to the pre-metastatic niche before the arrival of tumor cells	Lung carcinoma and melanoma	^10,77^
	TGF-α	Promote cancer cell invasion, prolapse from the primary site and blood vessels, thereby promoting tumor metastasis	Breast cancer	^484,485^
	HGF	Activation of the c-MET-ERK1 / 2-ELK1 pathway up-regulates ETV1 expression and up-regulates metastasis-related genes (PTK2 and MET) to promote metastasis	Hepatocellular carcinoma and pancreatic cancer	^486,487^
	TNF-α	TNF-α-TNFR1 activated inflammatory macrophages produce a large amount of VEGF-C, thereby coordinating the activation of VEGFR3 and promoting inflammation and lymphangiogenesis	Lung carcinoma	^488^
	IL-6	Through the trans receptor on the surface of the primary tumor cells, it promotes the metastasis and invasion of CTC and induces its stemness	Breast cancer	^48,143^
	IL-8	Induce tumor cells to mesenchymal phenotype transformation, migration and invasion ability enhancement; induce tumor-resident macrophages to polarize into tumor-associated macrophages	Gastric cancer	^489^
	IFN-γ	Inducing tumor antigen loss, upregulating PD-L1 in tumor cells, and recruiting myeloid-derived suppressor cells and tumor-associated macrophages to the tumor and metastatic sites	Breast cancer	^490^
	TGF-β	Accumulates TAMs and Treg cells in the pre-metastatic niche; induction of S100A8 and S100A9 release promotes pre-metastatic niche epithelial-mesenchymal transition	Colorectal cancer	^491,492^
	Bcl-2	Synergistic proto-oncogene ras inhibits tumor cell apoptosis, promotes tumor cell growth and metastasis	Prostate cancer	^6,493^
	IL-10	Upregulating the expression of PD-L1 in monocytes, reducing CD8 + T cell infiltration and related anti-tumor immunity in the context of metastasis	Colorectal cancer	^494^
	IL-1β	Inducing overexpression of SLC7A11 upregulates PD-L1 and CSF1 through the αKG/HIF1α axis, thereby promoting TAM and MDSC infiltration, which in turn facilitates cancer metastasis	Hepatocellular carcinoma	^495^
	S100A8/A9	Binding with TLR4, RAGE, CD147, MCAM, and NPTN triggers the MAPK, NF-κB, and Akt pathways, stimulating tumor initiation, progression, and metastasis	Lung carcinoma	^496^
	CCL2	Promoting the differentiation of monocytes into metastasis-associated macrophages and the formation of pre-metastatic niche, accelerating the colonization and growth of metastatic tumor cells	Prostate cancer and Lung carcinoma	^497^
Extracellular vesicles	miR-10b	Regulating the polarization of M2 macrophages to promote tumor cell invasion, metastasis, and epithelial-mesenchymal transition processes	Lung carcinoma	^498^
	miR-21	Targeting SMAD7 activates the TGF-β/Smad pathway to induce mesothelial-to-mesenchymal transition, promoting cancer metastasis	Gastric cancer	^499,500^
	miR-155	Through the STAT3 signaling pathway, it promotes the expression of EMT transcription factors Twist, Snail, Zeb1, Zeb2, and Slug, enhancing cancer cell migration	Oral squamous cell carcinoma	^501^
	miR-494	Downregulating BMAL1 transcriptionally promotes GPAM expression in an EZH2-dependent manner to facilitate glycerolipid metabolism	Hepatocellular carcinoma	^502^
	miR-373	Upregulating YAP1 expression and binding with ZEB1, the complex facilitates metastasis through the ZEB1/YAP1-ITGA3 signaling axis	Pancreatic cancer	^503,504^
	miR-105	ZO-1 degradation promoted CTCs to break through the vascular barrier into lung parenchyma	Breast cancer	^505^
	miR-122	Inhibition of glucose uptake by pre-metastatic niche cells through downregulation of glycolytic enzyme pyruvate kinase, thereby increasing the nutritional supply to pre-metastatic niche in the lung	Breast cancer	^210^
	miR-520c	Suppressing the expression of the hyaluronan (HA) surface receptor CD44 protein to promote tumor cell invasion and metastasis	Breast cancer	^504^
	miR-934	Induction of M2 macrophage polarization and secretion of CXCL13 induces the formation of pre-metastatic niche	Colorectal cancer	^131^
	miR-519a-3p	Activation of the MAPK/ERK pathway induces M2-like polarization of macrophages to promote angiogenesis and formation of pre-metastatic niche in the liver	Gastric cancer	^135^
	miR-181a-5p	Activation of CCL20 secreted by hepatic stellate cells activates the CCL20/CCR6/ERK1/2/Elk-1/miR-181a-5p positive feedback loop leading to reprogramming of TME	Colorectal cancer	^506^
	miR-25-3p	Targeting KLF2 and KLF4 to regulate endothelial cells promotes vascular permeability and angiogenesis	Pancreatic ductal adenocarcinoma	^140^
	miR-4508	Targeting RFX1-IL17A-p38 MAPK-NF-κB signaling pathway promotes lung PMN formation	Hepatocellular carcinoma	^507^
	miR-135a-5p	Initiation of the large tumor suppressor kinase 2-yes-associated protein-matrix metalloproteinase 7 axis	Colorectal cancer	^508^
	miR-92a-3p	Induction of macrophage immunosuppressive phenotypic differentiation and increased PD-L1 expression by inhibition of the PTEN-ERK signaling pathway	Gastric cancer	^509^
	miR-151a-3p	Promoting hepatic stemness-permissive niche formation by modulating SP3 and TGF-β1 pathways in Kupffer cells	Gastric cancer	^510^
	miR-455	Targeting ZO-1 enhances vascular permeability and promotes metastasis	Nasopharyngeal carcinoma	^511^
	miR-106a	Targeting Smad7 and TIMP2 activates TGF-β pathway, induces MMT and accelerates ECM degeneration	Gastric cancer	^512^
	miR-378a-3p	Activation of the Dyrk1a/Nfatc1/Angptl2 signaling pathway in bone marrow macrophages induces osteolytic metastasis	Prostate cancer	^513^
	miR-203a-3p	Induction of macrophage M2 polarization secretes CXCL12 and promotes metastatic niche formation through the CXCL12/CXCR4/NF-κB signaling pathway	Colorectal cancer	^514^
	miR-374a-5p	Targeting ADD3 Regulates the Distribution of ZO-1 and Occludin in Endothelial Cells, Increases Vascular Permeability and Promotes LM	Non-small cell lung cancer	^515^
	miR-6750	Inhibition of angiogenesis and activation of macrophages toward M1 phenotype	Nasopharyngeal carcinoma	^516^
	miR-92a	Inhibition of the target SMAD7 enhanced TGF-β signaling in hepatic stellate cells	Lung cancer	^517^
	miR-221/222	Inhibition of SPINT1 expression activates liver hepatocyte growth factor	Colorectal cancer	^518^
	miR-3473b	Phagocytosis by lung fibroblasts and leads to NF-κB signaling activation	Lung cancer	^519^
	miR-3157-3p	Targeting TIMP/KLF2 regulates endothelial cells thereby promoting angiogenesis and increasing vascular permeability	Non-small cell lung carcinoma	^520^
	tRF-GluCTC-0005	Up-regulation of WDR1 activates hepatic stellate cells, which mediates infiltration of MDSCs to form PMNs	Pancreatic ductal adenocarcinoma	^521^
	ITGBL1	Activation of NF-κB signaling pathway activates IL-6 and IL-8 secretion by resident fibroblasts in metastatic organs.	Colorectal cancer	^522^
	Clathrin light chain A	Stabilizing BSG in endothelial cells to remodel pre-metastatic microvascular niches	Hepatocellular carcinoma	^275^
	LOXL2	Induce EMT and fibronectin production in premetastatic organs	Head and neck squamous cell carcinoma	^523^
	S100A11	MDSCs were recruited to pre-metastatic niche	Osteosarcoma	^524^
	LINC00482	Modulation of the miR-142-3p/TGF-β1 axis induces microglia M2 polarization and affects the pre-metastatic niche	Non-small cell lung cancer	^291^
	ADAM17	Targeting vascular endothelial cells induces vascular leakage mediating pre-metastatic niche formation	Colorectal cancer	^525^
	Nidogen 1	Enhanced angiogenesis and pulmonary endothelial permeability	Hepatocellular carcinoma	^526^
	ANGPTL1	Reprogramming Kupffer cells and reducing MMP9 expression	Colorectal cancer	^231^

Inflammatory chemoattractant proteins S100A8 and S100A9, induced by various TDSFs like VEGF-A, TNF-α, and TGF-β, facilitate the infiltration of Mac1+ myeloid cells into the lung PMN.^65^ Moreover, VEGF emanating from the primary tumor modifies the lung microenvironment in the pre-metastatic phase by inciting an inflammatory response and producing PGE2, a pivotal element in the establishment of the PMN.^40^ Prior research has demonstrated that TDSFs can attract MDSCs to the PMN. through the S1PR1-STAT3 signaling pathway, resulting in a microenvironment suitable for tumor growth before the arrival of CTCs.^111^ Additionally, CCL2 can recruit TAMs and Tregs, stimulating angiogenesis, inhibiting immune cell function, and promoting PMN establishment in the lung.^112^ Within the PMN, host stromal cells might enhance the expression of inflammatory factors following stimulation by TDSFs. Subsequently, BMDCs or immune cells are attracted to the PMN, further augmenting the secretion of inflammatory factors.^11^ These TDSFs play a crucial role in the exchange of information within the PMN through different signaling pathways.

#### Extracellular vesicles

EVs are lipid bilayer-encased entities that are extruded from the cellular membrane and transport nucleic acids and proteins. EVs are commonly categorized into several subtypes, which encompass exosomes, microvesicles, apoptotic bodies, oncosomes, and megasomes.^113^ The formation of the PMN arises from the intricate interplay between TDSFs and EVs.^114^ In the microenvironment of PMN, we put our focus on tumor-derived exosomes and microvesicles. Exosomes, which are vast membranous structures, transport a diverse array of substances to their surroundings, including proteins, sugars, lipids, metabolites, RNA, and DNA, impacting the formation of the PMN.^15,115,116^ Tumor-derived EVs promote the establishment of an immunosuppressive PMN in target organs by impairing NK cell function and hindering DC maturation.^117,118^ For instance, exosomes from ETS1-overexpressing ovarian cancer cells specifically promote omental metastasis by mediating the oncogenic influence of macrophages.^119^

Tumor-derived exosomes may encapsulate a diverse array of proteins, including integrins and carcinogenic proteins.^120^ Studies have shown that the proteins contained in exosomes in different cancer types are highly heterogeneous.^121^ Breast cancer stem cells induced by Lin28B are a major source of exosomes containing low levels of let-7s, which are essential for establishing an immunosuppressive PMN and promoting lung metastasis in breast cancer.^98^ Furthermore, exosomes originating from pancreatic cancer, abundantly laden with macrophage migration inhibitory factor, allure macrophages and catalyze the establishment of the PMN in the liver, thereby augmenting the hepatic metastatic load.^35^ However, in the liver, colorectal cancer-derived exosomes can carry TGF-β1. It can not only induce hematopoietic stem cells to differentiate into tumor-associated fibroblast phenotypes, but also recruit MDSCs to PMN and inhibit the cytotoxicity of NK cells by downregulating the expression of NKG2D. Eventually an immunosuppressive PMN is formed.^122^ However, how does cancer control organ-specific metastasis? What is the mechanism? Previous studies have shown that integrins expressed on exosomes can determine adhesion to particular cell types and ECM molecules within designated organs. Exosomes expressing ITGαvβ5 selectively adhere to Kupffer cells, whereas those expressing ITGα6β4 and ITGα6β1 target resident lung fibroblasts and epithelial cells.^123^ Exosomes expressing integrin α5 can specifically induce the PMN of bone formation.^124^ Overall, these findings clearly illustrate the fundamental role of integrins in organ-specific metastasis. Furthermore, tumor-derived exosomes facilitate immunosuppression by reprogramming glycolysis and lipid metabolism in interactions with various cells, thereby aiding in PMN formation.^27,125^ Investigating the mechanisms of exosomes and metabolic reprogramming can not only help in exploring the PMN but also identify novel therapeutic targets for cancer progression.^126^ In addition to cell-cell interactions, EVs can induce the shedding of CTCs at the primary tumor site by activating the Wnt and PTEN pathways in the early stages of metastasis. This promotes epithelial-mesenchymal transition (EMT) and initiates the formation of the PMN.^127^

Similarly, exosomes can also transport RNA, including microRNA (miR) and non-coding RNA.^128,129^ These exosomal RNA are transported to the PMN, which not only mediates intercellular communication, but also serves as a biomarker to guide diagnosis.^130^ Studies have demonstrated that tumor-derived exosomal miR-934 can induce M2 macrophage polarization by suppressing PTEN expression and activating the PI3K/AKT signaling pathway.^131^ Polarized macrophages not only facilitate the formation of the PMN but also secrete CXCL13 to activate the CXCL13/CXCR5/NFκB/p65/miR-934 positive feedback loop, thereby accelerating the progression of cancer metastasis.^132,133^ Exosomal miR-519a-3p derived from gastric cancer can be transported to the liver where it targets DUSP2, activating the MAPK/ERK pathway and leading to M2-like polarization of macrophages. These polarized macrophages within the liver further promote angiogenesis, thus contributing to the establishment of an intrahepatic PMN.^134,135^ EVs encapsulating circular RNA can also promote cancer metastasis.^136^ In renal cell carcinoma, EVs containing circEHD2 activate fibroblasts within the PMN, promoting their transformation into cancer-associated fibroblasts (CAFs) and thereby facilitating renal cell carcinoma metastasis.^137^ In summary, EVs from primary tumors can aid in PMN formation by promoting immunosuppression and lymphangiogenesis.^98,138,139^ The mechanisms vary across different types of tumors.^140^ With more in-depth research on EVs, their role as a prognosis and biomarker for cancer metastasis treatment is becoming increasingly significant.

#### Other molecular components

The formation of a PMN also necessitates the involvement of molecular components. such as chemokines and inflammatory factors. Studies have shown that elevated levels of interleukin-6 (IL-6) can not only activate the JAK/STAT3 pathway, but also achieve feed-forward autocrine elevation of IL-6 leading to a vicious cycle.^141^ Eventually lead to angiogenesis, inflammation and immunosuppression, all of which provide convenience for PMN.^142,143^ Similarly, stromal cells expressing the chemokine CCL2 receptor can recruit inflammatory monocytes and metastasis-associated macrophages, promoting the exudation of CTCs in the primary tumor site.^144^ Metastatic macrophages contribute to immunosuppression in the PMN, aiding the arrival and colonization of CTCs. In summary, apart from molecules originating from the primary tumor, other cellular or matrix-derived components also contribute to the formation of the PMN. This proves that PMN is not only regulated by primary tumors but also has obvious heterogeneity in different tumor types.^145,146^

## Chronological insights into the mechanism of pre-metastatic niche formation

**Fig. 3:**
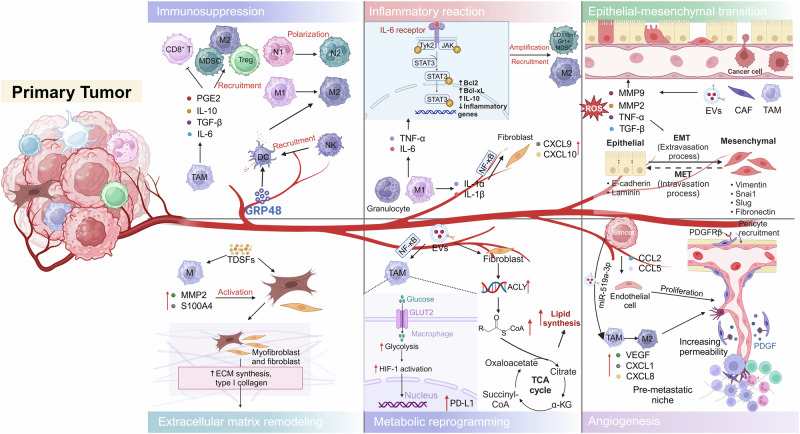
Chronological insights into the mechanism of pre-metastatic niche formation: Deciphering the steps leading to metastasis. The primary mechanisms underlying the formation of the pre-metastatic niche involve epithelial-mesenchymal transition, immunosuppression, extracellular matrix remodeling, metabolic reprogramming, vascular permeability and angiogenesis. The pre-metastatic niche is fostered by factors and cells such as tumor-derived soluble factors and BMDMs released by the primary tumor, which facilitate its establishment and promote the colonization of tumor cells

The formation of the PMN is recognized as a complex, multi-stage process involving multiple cells and molecules. This process, leading from the inception and progression of the PMN to the establishment of metastases, is divided into three stages: (a) The primary tumor releases various cellular and molecular components to remotely control PMN. (b) PMN microenvironment is remodeled to be suitable for CTC colonization through various mechanisms. (c) CTCs target specific organs for infiltration and colonization to form metastases.^147,148^ According to the chronological and spatial order, we show the relevant molecular mechanisms of PMN from onset, development to formation (Fig. 3).

### Epithelial-mesenchymal transition

The EMT is pivotal in allowing tumor cells to disengage from the primary tumor site and migrate to remote areas within the body. This process endows them with invasive and metastatic capabilities, thereby driving malignant progression.^83,149^ During this process, initially proliferating epithelial-like cancer cells undergo phenotypic changes, shedding their cell-to-cell adhesion characteristics and adopting a fibroblast-like phenotype characterized by enhanced invasiveness and migratory capabilities, thereby facilitating the dissemination of metastatic cells. However, the colonization of CTCs requires some epithelial characteristics to restore metastatic growth. Before metastasis, the niche needs to undergo mesenchymal epithelial transformation to promote the infiltration and colonization of tumor cells.^150,151^ Numerous cytokines and signaling pathways, such as TGF-β, Wnt, Notch, and Hedgehog, are involved in regulating EMT.^152,153^ Tumor-derived exosomes can carry TGF-β, hypoxia-inducible factor 1α (HIF1α) and β-catenin, which are factors that promote EMT.^154,155^

TAMs contribute to this process by generating ROS, which in turn recruit CAFs and activate matrix metalloproteinases.^46^ Additionally, TAMs secrete the pro-inflammatory cytokine TNF, which activates NF-κB signaling in stromal cells and cancer cells, leading to the upregulation of SNAI1 expression. Additionally, in reaction to both intrinsic and extrinsic oxidative stress, CAFs bolster tumor growth and augment cancer cell EMT by secreting growth factors and enzymes that degrade the ECM.^156^ Pericytes contribute to the establishment of the distant PMN. Within this niche, tumor-derived soluble factors and MDSCs influence pericytes, resulting in a diminished population of pericytes and compromised or absent linkages between endothelial cells and the basement membrane, resulting in the ability of CTCs to compete with pericytes to infiltrate the basement membrane to achieve colonization.^157,158^

Recent findings suggest that TNF-α produced by TAMs can stabilize Snail through the NF-kB signaling pathway, while TGF-β produced by TAMs induces the expression of Snail and ZEB1 via activation of the β-catenin pathway, among other mechanisms. Furthermore, mesenchymal cells secrete GM-CSF, which activates TAMs and induces CCL18 production, thereby promoting a positive feedback loop for EMT.^34^ TAMs actively participate in the formation of the PMN and remodeling of the tumor ECM by secreting proteolytic enzymes (including MMP2 and MMP9) and matrix-associated proteins.^159^ Additionally, pro-inflammatory factors can further facilitate the establishment of the PMN. For instance, heightened concentrations of IL-6 and IL-1β can activate ITGB1, augmenting the adherence of bone marrow cells to endothelial cells and inducing EMT to facilitate metastasis.^143,160^ Subsequently, at the metastatic site, TAMs secrete IL-35, reversing EMT and enhancing tumor cell colonization through the JAK2-STAT6-GATA3 signaling pathway.^161^ The PMN can recruit bone marrow-derived CD11b^+^ GR1^+^ myeloid progenitor cells and secrete versican in a paracrine manner, which can induce diffuse stromal tumor cells recruited to PMN to undergo mesenchymal-to-epithelial transformation to form metastases.^162^

At present, many studies have shown that in vitro factors can also induce the formation of PMN through certain mechanisms. For example, the accumulation of nicotine in the lungs of smoking patients can activate neutrophils to secrete the glycoprotein lipocalin-2 (LCN2). This promotes the paracrine-mediated functional reversal of mesenchymal cancer cells into epithelial phenotypes, enhancing the infiltration and growth of cancer cells.^163,164^ Fascinatingly, alcohol consumption may instigate the release of pro-inflammatory cytokines. Increased concentrations of these cytokines during the initial phases of cancer can function as early paracrine signals, fostering EMT to augment metastatic potential.^165^ Similarly, capsaicin and cold exposure may enhance the interaction between LAMC2 and ITGB1, activate p-FAK in PMN and up-regulate snail expression. Eventually, the process of EMT is accelerated and early PMN is formed.^166,167^ In summary, EMT is not only the initial step in the formation of PMN but also an important mechanism by which CTCs invade and colonize PMN to achieve metastasis.

### Immunosuppression

Immunosuppression constitutes a pivotal stage in the formation of the PMN, as each stage of distant tumor metastasis is susceptible to immune surveillance.^168^ The PMN is charaterized by a significant infiltration of immune cells, comprising diverse subsets such as macrophages, granulocytes, MDSCs, and T lymphocytes.^15,169^ Within the TME, immune cells undergo polarization towards a pro-invasive and immunosuppressive phenotype, thus facilitating tumor progression.^170^

For example, metastatic lesions within the brain parenchyma consist of a combination of resident microglia and infiltrating BMDMs, which together help form the PMN.^171^ TAMs and their secreted factors are implicated in the induction of EMT in various cancer contexts.^172^ TAMs release cytokines including IL-6, TGF-β, WNT, and pleiotrophin, which trigger their respective signaling pathways in cancer stem cells, thereby promoting their proliferation and differentiation.^173^ Furthermore, cancer stem cells possess the capability to co-migrate with macrophages to metastatic sites, contributing to the establishment of an immunosuppressive milieu within the PMN, thereby facilitating the survival and proliferation of CTCs.^174,175^ Moreover, NLRP3 signaling in macrophages can enhance the differentiation of CD4 + T cells into tumor-promoting T helper type 2 cells, Th17 cells, and Tregs populations, simultaneously inhibiting Th1 cell polarization and diminishing the activation of cytotoxic CD8 + T cells.^176,177^

An in vivo study revealed that tumor-secreted GRP78 interacts with hepatic DCs and macrophages within the metastatic niche, prompting the development of tolerogenic phenotypes. This interaction impedes the recruitment and activation of NK cells, thereby promoting the establishment of a PMN favorable for tumor cell colonization, invasion, and metastasis.^178,179^ Additionally, the presence of soluble GRP78 in the liver has been found to impact the motility of CX3CR1+ cells, including DCs and macrophages, resulting in immunosuppression and liver metastasis. Furthermore, studies have revealed the upregulation of SIGLEC15 expression in lymph node PMN, with SIGLEC15 predominantly expressed on M2 macrophages. These findings suggest that SIGLEC15 exerts an immunosuppressive influence on the pre-metastatic lymph nodes and underscore the role of macrophages in the development of an immunosuppressive PMN.^180^

Recent investigations have examined the impact of Oxaliplatin on the pre-metastatic microenvironment. A notable decrease in the number of macrophages and T cells was observed in the livers of colorectal cancer patients receiving Oxaliplatin chemotherapy. However, macrophages exhibit a more immunosuppressive phenotype in Oxaliplatin-primed livers, which may contribute to liver metastasis.^181^ In pancreatic cancer, initiation of the PMN involves TEVs triggering Kupffer cells to produce TGF-β and hepatic stellate cells to produce fibronectin. This results in the mobilization of BMDMs, which facilitates the creation of an immunosuppressive milieu within the PMN.^182^ EVs can additionally amplify the immunosuppressive capacities of macrophages by elevating the expression of PD-L1 and cytokine secretion through STAT3 signaling.^183^ Exosome-derived MIF has been demonstrated to activate Kupffer cells to release fibronectin and TGF-β, resulting in the formation of a fibrotic and immunosuppressive microenvironment at the metastatic site. These secreted factors assist in recruiting BMDMs to the hepatic PMN. The exosome regulation pathway in PMN can promote the immunosuppression of pancreatic ductal adenocarcinoma and accelerate the progression of metastasis.^25^

Chronic inflammation is pivotal in tumor progression and metastasis. Macrophages resident in premetastatic sites actively contribute to establishing an immunosuppressive microenvironment with inflammatory characteristics. For instance, in Lewis lung carcinoma, the tumor-derived proteoglycan versican can stimulate host macrophages to assume an M1 phenotype, culminating in the secretion of TNF-α and the creation of an inflammatory microenvironment.^184^ Furthermore, Chang et al. showed that IL-6 secreted by advanced-stage invasive breast cancer cells can activate STAT3 via JAK signaling, affecting both the tumor cells and the surrounding stromal cells. This activation further regulates the expansion of MDSCs marked by CD11b + /Gr1+ and the infiltration of macrophages (CD11b + /F480 + ) in distant organs like the lungs, ultimately fostering an inflammatory immunosuppressive microenvironment at pre-metastatic sites.^185^ It is well-established that IL-1β is primarily produced by tumor-associated granulocytes and macrophages. Elevated levels of IL-1α/β induce phenotypic changes in lung fibroblasts, triggering the production of chemokines CXCL9 and CXCL10 via NF-κB signaling, thus promoting the development of an inflammatory PMN.^145^ Studies have shown that Gr1+ inflammatory monocytes expressing CCR2 (the receptor for CCL2) are drawn to the pre-metastatic lung through CCL2 secretion by both tumor and stromal cells. These monocytes subsequently differentiate into TAMs in the MMTV-PyMT breast cancer mouse model, promoting the growth of metastatic cells.^144^ Furthermore, irrespective of tumor type and organ location, Tumor-derived EVs can be intercepted by macrophages in the PMN and hematopoietic stem cells in the liver, prompting the initiation of an inflammatory reaction marked by increased expression of diverse cytokines, chemokines, and regulatory genes.^186^ Mac1+ macrophages, a specific subset of macrophages, play an essential role in promoting the formation of the PMN under inflammatory conditions, thus aiding the migration of primary tumor cells to secondary lung sites.^187^ Thus, the creation of an inflammatory, immunosuppressive microenvironment at a secondary site—either preceding or coinciding with the arrival of CTCs—is imperative for the successful implantation, survival, and expansion of tumor cells within the PMN.

### Extracellular matrix remodeling

The ECM plays a pivotal role in promoting tumor metastasis. The four key characteristics of tumor metastasis, including motility, regulation of the microenvironment at the invasive site, plasticity, and establishment of residence within the invasive tissue, are closely intertwined with the ECM.^188,189^ The ECM that enables the priming of the primary tumor for metastasis to a remote location, thereby creating a microenvironment supportive of tumor growth, is referred to as the “pre-metastatic ECM”. This pre-metastatic ECM plays a critical role in establishing a favorable environment for tumor metastasis by either reorganizing or modifying the existing ECM structure or by inducing local ECM secretion. The process of ECM remodeling is highly significant in facilitating the settlement of CTCs within the PMN.^24,189,190^

Secretory factors emanating from the primary tumor can influence the expression of ECM structural proteins, ECM-degrading enzymes, and ECM-processing proteins within the PMN, thereby facilitating ECM remodeling and contributing to the formation of the PMN.^191,192^ Studies have shown that PAD4, secreted by colorectal cancer cells, can induce the citrullination of type I collagen in the liver, thus enhancing the adhesion of disseminated tumor cells to hepatic tissue and aiding the metastasis of colorectal cancer cells to the liver.^193^ TAMs exhibit the expression and secretion of various membrane-associated proteins that contribute to the degradation of collagen fibers in the ECM. These proteins include matrix metalloproteinases, cysteine-rich acidic secretory proteins, and tissue proteases.^194^ Once collagen fibers are degraded, TAMs play a role in regulating the turnover of collagen fragments through processes such as phagocytosis and lysosomal degradation mediated by proteases. In models of lung adenocarcinoma and breast cancer, a distinct subset of TAMs has been found to express fibroblast activation protein (FAP)-α, serving dual roles as a signaling molecule for CAFs and as a collagenase. Furthermore, heme oxygenase (HO)-1 has been implicated in the process of ECM remodeling.^195,196^

Studies have demonstrated that in CCR2-DTR mice with pre-metastatic lungs, the inhibition of inflammatory monocyte recruitment through the CCL2-CCR2 axis resulted in a significant reduction in the expression of MMP9 and the extravasation of cancer cells. This suggests that the recruitment of inflammatory monocytes by the primary tumor, followed by macrophages, is instrumental in the upregulation of MMP9 expression within the pre-metastatic lung niche. Notably, pre-invasive macrophages can create microchannels for cancer cell invasion through a combination of protease-mediated ECM remodeling and physical forces exerted on the collagen matrix.^30^ A plethora of research has substantiated the direct participation of TAMs in fostering the development of tumor niches and modifying the composition of the tumor ECM via the secretion of proteolytic enzymes such as MMP-2 and MMP-9, alongside matrix-associated proteins.^197^ In a mouse model of colorectal cancer, TAMs were found to contribute to the deposition, crosslinking, and alignment of ECM collagen fibers, suggesting that the accumulation of ECM collagen in cancer cannot be solely attributed to CAFs.^198^ In addition, S100A4 secreted by macrophages can activate ERK signaling pathway, which plays a crucial role in the progression of pulmonary fibrosis.^199,200^ Activation of the ERK signaling pathway can trigger fibroblast activation and production of ECM proteins, thereby facilitating ECM remodeling within the PMN. This proves that normal stromal cells in secondary metastatic sites can also play a role.^201,202^ In general, ECM remodeling plays an important role in tumor cell invasion, migration, survival and immune escape during tumor metastasis. A thorough investigation into the mechanisms and regulation of ECM remodeling could unveil novel targets and strategies for cancer therapy, which is helpful for blocking the metastasis and diffusion of tumors and improving the therapeutic efficacy.

### Metabolic reprogramming

Metabolic reprogramming is crucial in establishing PMN.^203^ First, it provides the required growth and migration energy for tumor cells by adjusting the metabolic pathway, enabling them to actively involvement in PMN formation.^204^ Second, metabolic reprogramming enables tumor cells to adapt to the challenging conditions in the TME, including hypoxia and low nutritional status, to maintain their survival and proliferation ability and create favorable conditions for the formation of PMN. However, metabolic reprogramming can also affect the activity of intracellular signaling pathways and regulate cell proliferation, migration and invasion, thereby facilitating tumor cell growth and dissemination within the PMN.^205–207^

Studies have shown that exosomes derived from tumors can induce macrophages to adopt an immunosuppressive phenotype, characterized by heightened expression of PD-L1. This transformation is facilitated through NF-kB-dependent, glycolytic-dominant metabolic reprogramming. Tumor-derived exosomes induce NF-kB activation through TLR2 signaling, leading to the activation of HIF-1α/GLUT-1 and increased glucose uptake by macrophages.^208^ Additionally, NOS2/NO is utilized to inhibit mitochondrial oxidative phosphorylation. This metabolic shift enhances glycolysis and diverts pyruvate towards lactic acid production. Newly synthesized lactic acid can in turn activate NF-κB, further elevating PD-L1 expression and sustaining an intensified glycolytic phenotype. These discoveries indicate that tumor-derived exosomes, via glycolytic metabolism, can reprogram macrophages and play a role in establishing an immunosuppressive milieu within the PMN.^27,209^ Besides that, primary tumor-derived miR-122 can also inhibit glucose uptake and utilization in cells within the PMN by down-regulating glycolytic enzymes and pyruvate kinases, thereby achieving energy metabolism reprogramming to promote metastasis.^210^

Similarly, cancer-derived exosomes can also interact with the stromal cells in PMN to achieve metabolic reprogramming. Cancer-derived exosome HSPC111 is capable of not only inducing the transformation of fibroblasts into CAFs within the PMN, but also phosphorylating ACLY to elevate acetyl-CoA levels and influence the lipid metabolism of these CAFs.^125^ This not only enhances the immunosuppressive milieu of the PMN but also facilitates the proliferation and metastasis of cancer cells through heightened expression and activity of ACLY.^211–213^ Despite the scarcity of research on metabolic reprogramming within the PMN, the significance of metabolism in cancer metastasis is undeniable. An in-depth study of the role and significance of metabolic reprogramming in the formation of PMN will help to reveal the mechanisms underlying tumor metastasis, offer critical insights for identifying novel therapeutic targets and strategies, and provide new breakthroughs and hope in the field of tumor treatment.

### Vascular permeability and angiogenesis

Angiogenesis and vascular permeability are pivotal in the establishment of the PMN. Because neovascularization can provide sufficient oxygen and nutrition for the PMN, it also provides an appropriate excretion channel for the PMN. Vascular permeability affects the exchange of cells and molecules in the PMN.^214–216^ Increased vascular permeability contributes to the entry and exit of cells and their interaction with the surrounding environment, and promotes the growth and diffusion of the pre-metastatic niche.^217,218^

Long non-coding RNA (lncRNA) originating from CAFs are pivotal in the establishment of the PMN.^219,220^ The lncRNA SNHG5 increased the stability of ZNF281 mRNA in a m6A-dependent manner by recruiting IGF2BP2 in primary tumors. Subsequently, ZNF281-induced upregulation of CCL2 and CCL5 can stimulate P38MAPK signaling in endothelial cells within the PMN, facilitating angiogenesis, vascular permeability, and the establishment of the pre-metastatic microenvironment.^221^ TAMs play a crucial role in angiogenesis through the secretion of VEGF. A prior xenograft model investigation revealed that pancreatic cancer cells provoke TAM-mediated angiogenesis. This is achieved through the secretion of interleukin-35 (IL-35) by pancreatic cancer cells, which recruits TAMs and activates their secretion of CXCL1 and CXCL8, thereby stimulating angiogenesis.^222^ Perivascular macrophages have been found to induce vascular permeability and metastasis in the brain and lung through the secretion of tenascin C, nitric oxide (NO), and TNF.^223,224^ Previous studies have also reported that CECR1 is a molecule involved in regulating TAM polarization. Overexpression of CECR1 significantly upregulated the expression of platelet-derived growth factor subunit B (PDGF-B), a pro-angiogenic gene. The receptor for PDGF-B, PDGFR-β, is predominantly expressed on pericytes. CECR1 promotes the release of PDGFB from macrophages, thereby activating the PDGF-B/PDGFR-β pathway in pericytes, leading to the production of the Periostin protein and ultimately promoting angiogenesis.^225,226^ Cancer-derived exosome-mediated miR-25-3p can downregulate KLF2, KLF4, ZO-1, occludin, and Claudin5 while upregulating VEGFR2 prior to metastasis. KLF2 inhibits VEGFR2 to negatively regulate angiogenesis, and KLF4 maintains endothelial integrity by promoting ZO-1, occludin, and Claudin5.^227–229^ Low expression of KLF2 and KLF4 leads to vascular permeability and angiogenesis in the PMN.^140^

Furthermore, exosomal miR-519a-3p has the capability to trigger the MAPK/ERK pathway through targeting DUSP2, thereby promoting the M2 polarization of macrophages in the PMN. These polarized macrophages promote angiogenesis, thereby expediting the establishment of the PMN.^135,230^ However, research has demonstrated that exosomal ANGPTL1 can impede vascular permeability and delay the formation of PMN by reprogramming Kupffer cells and diminishing MMP9 expression.^231^ These studies underscore the critical role of angiogenesis and vascular permeability regulation in the establishment of the PMN, influencing its development and persistence, thus offering significant insights for the investigation and management of this niche.

## Landscape of pre-metastatic niche in metastasis organotropism

**Fig. 4:**
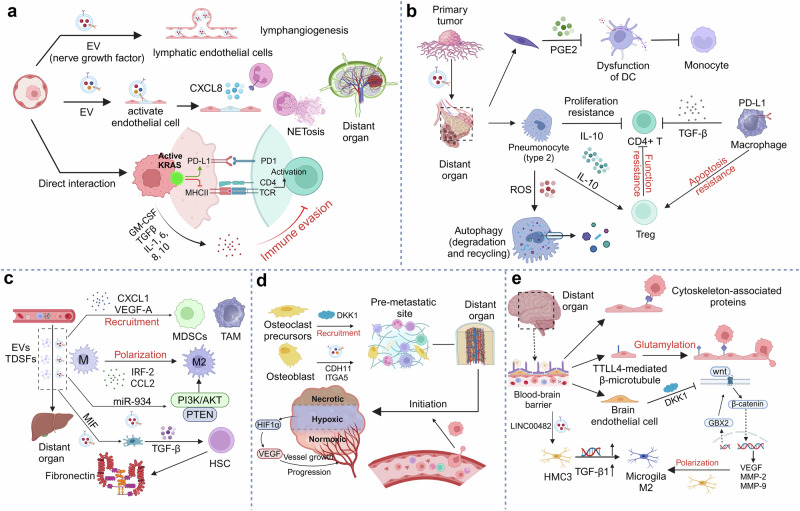
Landscape of pre-metastatic niche in various metastatic organs: Exploring the terrain for metastatic spread. **a** Lymph nodes primarily establish a pre-metastatic niche through mechanisms such as immune evasion and lymphangiogenesis. **b** In the lung, the pre-metastatic niche is formed mainly by inhibiting the local immune response, triggered by interactions between local immune cells and tumor-derived secretory factors. **c** Tumor cells interact with liver tissue by releasing factors and extracellular vesicles, leading to changes in the liver microenvironment. **d** In bone, the formation of a pre-metastatic niche involves stimulating osteoclasts, osteoblasts, and angiogenesis to provide growth space for metastatic cells. **e** At the primary site, tumor cells release factors and proteins that act on brain endothelial cells and astrocytes, crossing the blood-brain barrier and altering the immune environment of brain tissue to create conditions for pre-metastatic niche formation

One key aspect of metastasis is the establishment of a PMN in the target organ, which creates a favorable microenvironment for incoming cancer cells to colonize and proliferate. The composition of the PMN significantly influences the organ-specific spread of metastasis, determining the organotropism of cancer cells. Shared features in the establishment of the PMN across various organs encompass inflammatory reactions, angiogenesis, and immune modulation. However, cell types, microenvironments, and molecular signaling in different organs may result in variations in the methods of formation and influencing elements (Fig. 4). Studying these commonalities and differences is important for understanding organ-selective metastasis and developing targeted therapies.

### Lymph node

Lymph nodes are an important part of the lymphatic system. They are rich in vascular networks and immune cells, fostering a conducive ambiance for the proliferation and dissemination of cancer cells. Second, lymph nodes act as “filters” in the body. When cancer cells form metastatic niches in lymph nodes, they can avoid detection and attack by the immune system to a certain extent, increasing their chances of survival and diffusion. In addition, lymph nodes are strongly invasive, and cancer cells can destroy the tissue structure of lymph nodes and spread to surrounding tissues, further forming PMN.^232–234^

Factors originating from tumors, such as cytokines and exosomes, traverse through the lymphatic system to regional lymph nodes, substantially aiding in the formation of a PMN within the lymph node microenvironment. This prepares them as hospitable and supportive ecological niches for disseminated tumor cells.^235,236^ The PMN within the lymph node is distinguished by lymphangiogenesis and the restructuring of high endothelial venules, with lymphatic vessels serving as the primary conduit for tumor dissemination through the lymphatic system.^235,237,238^ Melanoma-derived EVs can be absorbed by lymph node endothelial cells and enrich nerve growth factor receptor (NGFR). Enrichment of NGFR not only induces the expression of phospho-ERK1 / 2 and endogenous NGFR, but also promotes lymphangiogenesis both in vitro and in vivo.^138^ Prior research has demonstrated that VEGF-C and VEGF-A are crucial in lymphangiogenesis.^239,240^ Tumor-derived EVs can interact with lymphatic endothelial cells (such as VCAM-1 and Ltb) in lymph nodes, resulting in immunosuppression during lymph node remodeling and facilitating the formation of the PMN.^139^

Diverse cellular interactions within the lymph node also play a role in the establishment of the PMN.^241^ The accumulation of tumor-derived EVs within the lymphatic endothelium leads to high expression of CXCL8, which promotes neutrophil influx and NET deposition in the PMN. NET deposition leads to a local microenvironment conducive to tumor growth.^72,242–244^ TAMs expressing podoplanin participate in recruiting and adhering these cells to lymphatic vessels expressing β1 integrin and GAL8. Once localized in the perilymphatic space, podoplanin-expressing macrophages directly promote ECM remodeling (independent of galectin 8 binding) and stimulate lymphangiogenesis and metastasis through both direct mechanisms (such as inducing lymphatic growth and cancer cell invasion) and indirect mechanisms (such as releasing VEGFC and VEGFD from the matrix).^245^ It has been demonstrated that the S1P receptor 1 (S1PR1) signaling pathway in macrophages plays a crucial role in promoting lymphangiogenesis through NLRP3-dependent secretion of IL-1β. In vitro studies have further revealed that macrophage-mediated lymphangiogenesis is initiated upon inflammasome activation, which necessitates the involvement of S1PR1 signaling and the production of IL-1β.^246^ In lymph nodes, DCs can induce SDF-1-induced premetastatic niche formation in COX-2/EP3 signaling pathway-dependent manner.^247^

In summary, the characteristics and advantages of lymph nodes as PMN are that they provide a suitable growth environment, occultation and invasiveness, which promotes the possibility of cancer cells forming PMN within lymph nodes and spreading further. Moreover, secreted factors and cells of multiple cancer origins can drain into the lymph nodes, and as a pre-metastatic outpost, the remodeling of lymph nodes creates a unique microenvironment for tumor cells and lymphatic endothelial cells, which is conducive to cancer metastasis.

### Lung

The lungs have unique characteristics as a PMN. A variety of cancers can metastasize to the lungs, including breast, colon, and prostate cancer.^248^ This is because the lungs are an important part of the systemic blood circulation and lymphatic system, and cancer cells may infiltrate the lungs from the primary site via the bloodstream or lymphatic system. Cancer cells may infiltrate the lungs from the primary site via the bloodstream or lymphatic system. The lungs provide a rich network of blood vessels and oxygen supply, which provides an optimal environment for the growth of cancer cells. In addition, the lungs have a large surface area and a rich volume of blood circulation, facilitating the colonization and proliferation of metastatic cancer cells within the lungs. The lungs also have unique microenvironments, such as low surface tension and strong gas exchange function in the alveoli, which provide suitable growth conditions for cancer cells.^14,249,250^

The importance of interactions between the ECM and immune cells in the formation of the PMN has attracted increasing attention.^251,252^ Recent investigations reveal that lung fibroblasts with elevated cyclooxygenase 2 (COX-2) expression can synthesize PGE2, which promotes the dysfunction of DCs and inhibitory monocytes. This proves that lung fibroblasts can reshape the immune environment of the PMN, thereby promoting cancer metastasis.^253^ Elevated GPX3 expression in alveolar type 2 epithelial cells can stimulate the release of IL-10 via hypoxia-inducible factor 1α (HIF-1α). This process inhibits CD4 + T cell proliferation while promoting the generation of Tregs, culminating in the creation of an immunosuppressive PMN.^254^ A large amount of exposure to particulate matter in the lungs can induce the production of ROS, thereby inducing autophagy in alveolar epithelial cells and leading to the degradation of TRIM37. Finally, the accumulation of TRAF6 protein is induced, which leads to the activation of NF-κB pathway and the increased production of chemokine production, and subsequently facilitates the development of a PMN via the recruitment of neutrophils.^255^ Breast cancer-educated alveolar macrophages have been shown to diminishes the number and maturation of lung DCs by modulating TGF-β expression, thus fostering an immunosuppressive milieu within the lung.^95^ Exposure to the β-adrenergic agonist isoproterenol has been observed to enhance CCL2 expression in lung stromal cells and increase CCR2 expression in monocytes/macrophages. This cascade recruits macrophages and fosters the development of a PMN in the lung.^256^ The aforementioned research underscores the critical role of stromal and immune cells in establishing the PMN within the lung.

Studies have shown that cancer immunotherapy can affect the progression of cancer metastasis.^257,258^ In melanoma, anti-PD-1 immunotherapy can activate the HSP70-TLR4 signaling pathway in lung epithelial cells. Activation of this pathway induces downstream Wnt5a-dependent release of G-CSF and CXCL5, thereby promoting myeloid granulocyte production and MDSCs recruitment into lung tissue.^259^ A recent study proposed that Cav-1 contained within exosomes derived from breast cancer can promote the formation of PMN by stimulating the expression of genes associated with formation of a PMN within lung epithelial cells and promotion of M2 polarization in lung macrophages.^41^ Furthermore, exosomes harboring Cav-1 can suppress the PTEN/CCL2/VEGF-A signaling pathway, thereby promoting M2 polarization and angiogenesis in lung macrophages.^42^ Factors derived from hepatocellular carcinoma cells upregulate IL-1β expression in alveolar macrophages. On one hand, IL-1β induces autocrine expression of MMP9 in alveolar macrophages. Conversely, IL-1β has been observed to augment the expression of SAA3 in alveolar epithelial cells, thereby attracting MMP9+ myeloid cells to the lungs. This process results in the creation of a permissive PMN that facilitates lung metastasis of hepatocellular carcinoma.^260^ In summary, the lung as a PMN has a unique microenvironment conducive to cancer growth, and these factors together promote the potential for a wide range of cancer cells to establish a PMN in the lung and spread further.

### Liver

The liver, serving as a PMN, is distinguished by its abundant blood supply and metabolic activities, immune evasion capabilities, profusion of cytokines and growth factors, and intricate anatomy. These factors collectively foster an environment conducive to the establishment of diverse cancer cell types in the liver and their subsequent dissemination.^261^ In the realm of colorectal cancer liver metastasis, increased YAP activity in fatty liver promotes cancer cell growth and creates an immunosuppressive microenvironment infiltrated by M2 macrophages that produce CYR61.^262^ High CD36 expression in tumor cells triggers metastasis through a distinct mechanism. CD36 is highly expressed on macrophages, and its expression is increased in tumor-associated metastasis-associated macrophages and BMDMs.^263^

Recent findings indicate that cells of colorectal carcinoma release VEGF-A, prompting TAMs within the primary tumor to generate CXCL1. This rise in CXCL1 within pre-metastatic liver tissue attracts CXCR2-positive MDSCs, establishing a PMN that significantly enhances the likelihood of liver metastasis.^83^ Exosomal CCL2 can bind to specific receptors (CCR2) to activate macrophage recruitment and shift the M1/M2 phenotype towards the M2 phenotype. Additionally, CCL2 can induce monocytes to migrate to the site of injury and activate them into macrophages. These findings imply that CCL2 may enhance the development of a PMN in the liver and aid in the seeding of CTCs.^264^ In an in vivo study, it was demonstrated that macrophage uptake of exosomal IRF-2 can promote the secretion of VEGFC, which participates in lymphatic network remodeling and contributes to the formation of PMN in sentinel lymph node metastasis of colorectal cancer.^265^ Exosomal miR-934 from CRC cells induces M2 macrophage polarization by downregulating PTEN expression and activating the PI3K/AKT signaling pathway. These polarized M2 macrophages can subsequently foster the creation of a PMN and facilitate liver metastasis in colorectal cancer by releasing CXCL13. CXCL13, in turn, activates a CXCL13/CXCR5/NFκB/p65/miR-934 positive feedback loop in CRC cells.^131^ Interestingly, impairment of the gut vascular barrier can result in bacterial dissemination to the liver, fostering the creation of a PMN and facilitating the recruitment of immune cells, such as macrophages.^266^ Exosomal ANGPTL1 has been identified to modulate the secretion profile of Kupffer cells, notably decreasing MMP9 expression through inhibition of the JAK2-STAT3 pathway, thus restoring vascular integrity in the liver PMN.^231^ Exosomal macrophage migration inhibitory factor derived from pancreatic cancer has been demonstrated to stimulate Kupffer cells to release TGF-β. This action subsequently prompts hepatic stellate cells to synthesize fibronectin, an essential component in establishing a PMN within the liver.^35^

Interestingly, dietary factors can also influence cancer metastasis.^267,268^ Research has indicated that prolonged intake of capsaicin compromises the integrity of the intestinal mucosal barrier. on the one hand, leads to bacterial movement and colonization of the liver, and on the other hand, bacterial aggregation of bile acid metabolism leading to an increase in secondary bile acids, which modulates the recruitment of NKT cells in the liver. All these factors contribute to the development of a PMN in colorectal cancer liver metastases.^269^ In addition, alcohol uptake is equally capable of remodeling the immunosuppressed liver microenvironment and shaping the PMN.^270^ The mechanism involves upregulation of IL-6 and its receptor expression, activation of the STAT3 signaling pathway and upregulation of downstream LCN2. Elevated LCN2 drives T cell depletion, neutrophil recruitment, and epithelial plasticity in cancer cells, leading to a liver immune evasive PMN.^271–273^ Taken together, these findings indicate that the liver as a PMN is characterized by its abundant blood supply and metabolic functions, immune evasion capacity, and abundant cytokines and growth factors, which together promote the potential for a wide range of cancer cells to form PMN in the liver and spread further.^274^ The formation of the hepatic PMN depends on complex interplays among cancer cells and the liver microenvironment, encompassing processes such as angiogenesis, immunosuppression, inflammatory responses, and remodeling of the ECM.^275,276^

### Brain

A variety of cancers can metastasize to the brain, such as breast cancer, lung cancer, and melanoma. This is due to the brain’s integral role within the central nervous system and has a unique blood-brain barrier and microenvironment that allows cancer cells to enter and grow in the brain through the blood or lymphatic system.^277^ In addition, the blood-brain barrier within the brain can limit drug penetration, making treatment difficult. The brain is also rich in neurons and glial cells, providing a suitable environment for cancer cells to grow.^278,279^ In addition, the brain has complex anatomical structures, such as the brain parenchyma and the leptomeninges/ventricular system, and the different central nervous system microenvironments provide opportunities for the proliferation and spread of cancer cells.^171^ Oweing to the existence of the blood-brain barrier, special mechanisms exist for cancer brain metastasis to form PMN.^280,281^ Platelet-derived von Willebrand factor (VWF) was found to aggregate to a similar extent before and after brain metastasis. This finding suggested that VWF fibers can contribute to pre-metastatic ecological niche formation.^282,283^ Cytoskeleton-associated proteins play an important role in the adhesion and migration of cancer cells in the endothelial cell layer of the blood-brain barrier.^284^ Tubulin tyrosine ligase like 4 (TTLL4)-mediated β-microtubule protein glutamylation in the brain increases the transport of multivesicular vesicles and leads to altered EV characteristics, enhancing the adherence of breast cancer cells to the endothelial cells of the blood-brain barrier, as well as augmenting the permeability of these endothelial cells, thereby aiding in the formation of a PMN within the brain.^285,286^

Microglia remove apoptotic debris by phagocytosis under normal physiological conditions and participate in the proper adjustment of neural circuits to promote CNS homeostasis.^287,288^ However, microglia are able to shape specific hypoxic PMN to fuel cancer cell colonization during cancer brain metastasis, but the exact mechanism is less clear.^289,290^ For example, metastatic lesions within the brain parenchyma consist of a combination of resident microglia and infiltrating BMDMs, which collectively contribute to the formation of the PMN.^172^ Microglia are also capable of M2 polarization as mononuclear macrophages in the CNS. LINC00482 transported by EVs derived from non-small cell lung cancer upregulates the expression of the miR-142-3p target gene TGF-β1 in HMC3 cells, thereby promoting microglial M2 polarization. Microglial M2 polarization promoted the formation of PMN, thus expediting metastasis.^291,292^ Similarly, exosomes of lung cancer origin induce brain endothelial cells to secrete Dkk-1. Dkk-1 acts as an inhibitor of the classical Wnt/β-catenin pathway and is able to convert M1-like microglia into M2-like microglia.^293,294^ This led to immunosuppression within the PMN of the brain, facilitating the colonization of CTCs in the brain.^295^ Extracorporeal factors can also trigger the formation of a PMN in the brain.^296,297^ For example, long-term smokers are able to activate N2 neutrophils via STAT3 in the brain. N2 neutrophils release the exosomal miR-4466, which promotes stemness and metabolic switching of tumor cells in the brain through the SKI/SOX2/CPT1A axis, contributing to the construction of PMN.^298,299^

Taken together, these findings indicate that the brain as a PMN is characterized by its unique blood-brain barrier and microenvironment, abundant neurons and glial cells, and complex anatomical structure, these factors collectively enhance the potential for the formation of a PMN by diverse cancer cells in the brain, facilitating subsequent dissemination. However, the current research on the premetastatic niche in the brain is relatively limited and requires further exploration in the future.

### Bone

Bone tissue has a rich blood supply and a growth environment, such as bone marrow, which enables cancer cells to infiltrate and proliferate within the bone via the bloodstream or lymphatic system. In addition, bone is rich in cytokines and growth factors, providing a suitable environment for cancer cells to grow. The complex anatomical structure of bone, including the bone marrow cavity and bone trabeculae, provides opportunities for the proliferation and spread of cancer cells.^300,301^ Moreover, the bone constitutes a principal site for metastatic targeting, with cancer cells proliferating within bone through the processes of bone resorption and formation. Numerous cancers, including breast, prostate, and lung cancer, are capable of metastasizing to bone.^302,303^

Osteoclast precursors (OPs) function in breast cancer bone metastasis.^304^ R- spondin 2 (RSPO2) and RANKL interact with the receptor LGR4 to up-regulate DKK1 through the Gαq and β-catenin signaling pathways. The high expression of DKK1 was able to promote the recruitment of OPs, which in turn facilitated the formation of the PMN.^305,306^ Additionally, osteoblasts can facilitate the establishment of a PMN in the bone, aiding in the colonization and survival of CTCs. Breast cancer-derived EVs carrying osteoblast cadherin (CDH11) and ITGA5 synergistically not only induce osteoblasts to form PMN, but also promote osteoblast recruitment to cancer cells and invasive spread.^124,307,308^ Breast cancer can also lead to osteolytic bone metastasis. Exosomes released by SCP28 breast cancer cells are pivotal in enhancing osteoclast differentiation and activation, alongside recruiting and reprogramming macrophages to aid in the development of a PMN.^309^ The hypoxic microenvironment of bone provides CTCs with a rather superior living environment. The release of hypoxia-inducible factor (HIF) not only promotes the spread of primary tumors to bone, but also promotes the activation of HIF signaling in bone-resident cells, facilitating the migration of tumor cells to bone as well as the generation of osteoclasts.^310^ Hypoxia acts not only on the primary tumor but also on bone metastases, advancing the metastasis and colonization of tumor cells.^311–313^ In addition, the formation of PMN in cancer relies on interactions among multiple factors such as tumor-derived exosomes, cytokines, and osteoblasts and osteoclasts.^314–316^

Taken together, bone as a PMN is characterized by its rich blood supply and growth environment, bone-resident cells, a multitude of cytokines and growth factors, complex anatomy, and a hypoxic microenvironment, which together promote the potential for a wide range of cancer cells to form a PMN in bone and spread further.

## Therapeutic approaches for pre-metastatic niche

**Fig. 5:**
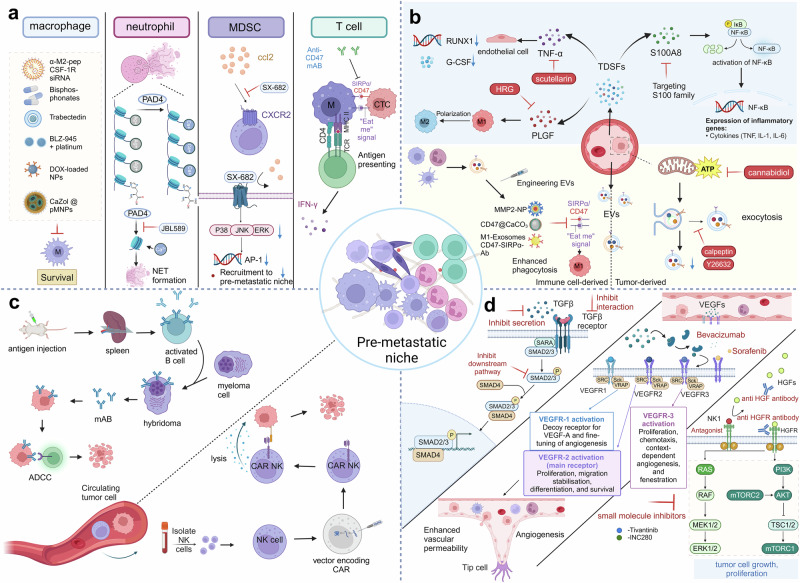
Therapeutic approaches for pre-metastatic niche: Strategies to disrupt the soil for metastasis. **a** Targeting cellular components in pre-metastatic niche formation. **b** Targeting molecular components in pre-metastatic niche formation. **c** Targeting circulating tumor cells. **d** Signaling pathways targeting pre-metastatic niche formation

The PMN orchestrated by the primary tumor establishes a conducive microenvironment for subsequent metastasis of tumor cells in secondary organs and tissues. A mature PMN may actively facilitate metastasis by promoting the extravasation of CTCs from blood vessels and attracting tumor cells to the niche. Current treatment strategies typically only intervene at specific stages of tumor metastasis, and there is currently no effective treatment strategy for the overall process of tumor metastasis.^24^ Targeting molecular components, cellular components, and related signaling pathways involved in the formation of the PMN to inhibit its formation may be a promising strategy for cancer treament (Fig. 5).^11,21,317^ In this section, we will further discuss this approach. At the same time, we have summarized our clinical trials for targeting cellular (Table 2) and molecular (Table 3) components involved in the formation of cancer metastasis.

**Table 2:** Clinical trials related to cellular components of pre-metastatic niche formation

Cellular type	Title	Disease or conditions	Interventions	Phase	Trial number
Macrophage	Phase 1 Study of Anti-Macrophage Migration Inhibitory Factor (Anti-MIF) Antibody in Solid Tumors	Metastatic Adenocarcinoma of the Colon or Rectum	Imalumab	Phase 1	NCT01765790
	Phase 1 Study of Macrophage Reprogramming Agent, exoASO-STAT6 (CDK-004), in Patients With Advanced Hepatocellular Carcinoma (HCC) and Patients With Liver Metastases From Either Primary Gastric Cancer or Colorectal Cancer (CRC)	Colorectal Cancer Metastatic to Liver, Gastric Cancer Metastatic to Liver	CDK-004	Phase 1	NCT05375604
	Phase I Pilot Study of RP1 in Primary Melanoma to Reduce the Risk of Sentinel Lymph Node Metastasis	Melanoma	Vusolimogene, oderparepvec	Early Phase 1	NCT06216938
Neutrophil	A Vaccine (MV-s-NAP) for the Treatment of Patients With Invasive Metastatic Breast Cancer	Metastatic Breast Adenocarcinoma	Oncolytic Measles Virus Encoding Helicobacter pylori Neutrophil-activating Protein	Phase 1	NCT04521764
	Study of Cabazitaxel Combined With Prednisone and Prophylaxis of Neutropenia Complications in the Treatment of Patients With Metastatic Castration-resistant Prostate Cancer (PROSPECTA)	Prostate Cancer	G-CSF, Ciprofloxacin, CABAZITAXEL, Prednisone	Phase 4	NCT01649635
MDSCs	SX-682 Treatment in Subjects With Metastatic Melanoma Concurrently Treated With Pembrolizumab	Metastatic Melanoma	SX-682, Pembrolizumab	Phase 1	NCT03161431
	Pilot Study of Nivolumab w/Ipilimumab or Relatlimab in Surgically Resectable Melanoma Brain Metastases	Metastasis to Brain	Nivolumab,Ipilimumab, Relatlimab	Early Phase 1	NCT05704933
	Depletion of Myeloid Derived Suppressor Cells to Enhance Anti PD-1 Therapy	Non Small Cell Lung Cancer Stage IIIB	Nivolumab, Gemcitabine	Phase 2	NCT03302247
	Ibrutinib and Nivolumab in Treating Participants With Metastatic Solid Tumors	Metastatic Malignant Solid Neoplasm	Ibrutinib	Phase 1	NCT03525925
	SX-682 and Nivolumab for the Treatment of RAS-Mutated, MSS Unresectable or Metastatic Colorectal Cancer, the STOPTRAFFIC-1 Trial	Metastatic Colorectal Carcinoma	CXCR1/2 Inhibitor SX-682, Nivolumab	Phase 1	NCT04599140
T cells	HER2-CAR T Cells in Treating Patients With Recurrent Brain or Leptomeningeal Metastases	Metastatic Malignant Neoplasm in the Brain	Chimeric Antigen Receptor T-Cell Therapy	Phase 1	NCT03696030
	Vemurafenib With Lymphodepletion Plus Adoptive Cell Transfer & High Dose IL-2 Metastatic Melanoma	Metastatic Melanoma	Lymphodepletion, Vemurafenib, High Dose Interleukin-2	Phase 2	NCT01659151
	A Study of OMP-313M32 in Subjects With Locally Advanced or Metastatic Solid Tumors	Metastatic Cancer	OMP-313M32, Nivolumab	Phase 1	NCT03119428
	A First-In-Human Phase 1 Trial of T-Cell Membrane-Anchored Tumor Targeted Il12 (Attil12)- T-Cell Therapy in Subjects With Advanced/ Metastatic Soft Tissue and Bone Sarcoma	Soft Tissue Sarcoma	Cyclophosphamide, attIL2-T cells	Phase 1	NCT05621668

**Table 3:** Clinical trials related to molecular components of pre-metastatic niche formation

Molecular type	Title	Disease or conditions	Interventions	Phase	Trial number
EGF	EGF-Depleting Therapy CIMAvax-EGF in Combination With Standard Therapy for RAS- and BRAF Wild-Type Metastatic Colorectal Cancer	Colorectal Cancer	Recombinant Human EGF-rP64K/Montanide ISA 51 Vaccine	Early Phase 1	NCT06011772
	EMB-01 in Patients With Advanced/ Metastatic Gastrointestinal Cancers	Metastatic Gastrointestinal Carcinoid Tumor	EMB-01	Phase 1/2	NCT05176665
FGF	Fulvestrant Plus Anlotinib in HR( + )/ HER2(-) Metastatic Breast Cancer With FGFR Mutation	Breast Cancer	Fulvestrant plus Anlotinib	Phase 2	NCT04936295
	Safety and Anti-Tumor Activity of TYRA-200 in Advanced Cholangiocarcinoma With Activating FGFR2 Gene Alterations (SURF201)	Metastatic Cholangiocarcinoma	TYR200	Phase 1	NCT06160752
	Evaluation of Infigratinib in Patients With Locally Advanced or Metastatic Gastric Cancer or GEJ Adenocarcinoma	Gastric Cancer	Infigratinib	Phase 2	NCT06206278
VEGF	A Phase II Trial of PD-L1 Therapy Combined With Anti-VEGF Therapy in Unresectable or Metastatic Melanoma	Clinical Stage III Cutaneous Melanoma AJCC	Atezolizumab, Bevacizumab	Phase 2	NCT04356729
HGF	Ficlatuzumab and Cetuximab in Recurrent/Metastatic Head and Neck Squamous Cell Carcinoma (HNSCC)	Squamous Cell Carcinoma of the Head and Neck	Ficlatuzumab, Cetuximab	Phase 1	NCT02277197
	To Evaluate the Safety, Tolerability, Pharmacokinetics and Antitumor Activity of YYB101 With Irinotecan, Patients Who Are Metastatic or Recurrent Colorectal Cancer Patients	Colorectal Cancer Metastatic	YYB101	Phase 1/2	NCT04368507
Extracellular Vesicles	Characterization of Extracellular Vesicles in Breast Cancer Patients	Breast Cancer	Blood sample	Observa-tional	NCT05798338
	Contents of Circulating Extracellular Vesicles: Biomarkers in Colorectal Cancer Patients (ExoColon)	Colorectal Cancer	Diagnostic test	Observa-tional	NCT04523389

### Targeting cellular components in pre-metastatic niche formation

#### Macrophages

Current therapeutic approaches aimed at macrophages involve inhibiting their recruitment, inducing apoptosis of TAMs, and reprogramming them towards an anti-cancer phenotype. The PMN is capable of recruiting macrophages, and the factors secreted by macrophages or their exosomes can directly or indirectly contribute to the metastasis and colonization of primary tumors. Resident macrophages lure inflammatory monocytes to the pre-metastatic site, where they transform into metastatic subsets of TAMs, thus facilitating the extravasation, seeding, and proliferation of metastatic cells.^317–319^ Therefore, immunotherapy targeting macrophages may potentially reduce the metastasis of primary tumors and the colonization of CTCs at distant sites.

The aggregation of macrophages within PMN creates an immunosuppressive microenvironment that facilitates the extravasation, seeding, and growth of metastatic cells.^320^ Strategies aimed at reducing the accumulation of macrophages in the PMN or blocking the recruitment of macrophages to the PMN may be beneficial for reducing the metastasis of primary tumors. The activation of the COX-1/TXA2 pathway in platelets has been demonstrated to enhance tumor cell adhesion to the endothelium, facilitate the recruitment of metastasis-promoting monocytes/macrophages, and aid in the formation of the PMN.^321^ Aspirin, a known inhibitor of the COX-1/TXA2 pathway, has been found to diminish the recruitment of monocytes/macrophages to the PMN.^322^ Therefore, selective inhibitors targeting the TXA2 pathway, such as picoramide, may represent a safe and promising treatment options for preventing metastatic disease.^323^

The spleen is acknowledged as a vital reservoir and origin of immune cells circulating in the body and infiltrating tumors. In a pancreatic cancer model, splenectomy has been shown to not only reduce the accumulation of TAMs in the PMN but also decrease the number of TAMs infiltrating the primary tumor.^320,324,325^ Nevertheless, the current literature regarding the impact of splenectomy on tumor progression and metastasis lacks conclusive findings. Therefore, further research is needed to investigate how splenectomy affects the aggregation of infiltrating inflammatory cells in the PMN, which can provide valuable insights for guiding future surgical methods and reducing the metastasis of primary tumors in patients.^326,327^ Research has shown that combining anti-ANG-2 therapy with low-dose metronomic chemotherapy effectively curtails the growth of metastases. This approach works by inhibiting inflammation and angiogenesis in the PMN through ANG-2 blockade and by preventing the recruitment of tumor-promoting CCR2+ Tie2-TAMs.^328^ Chemokines and CSF1 are known to play crucial roles in the recruitment of monocytes to tumors and the PMN, as well as in shaping their function within the TME.^329,330^ However, targeting chemokines alone or in combination has not yielded positive clinical outcomes, leading to the discontinuation of this strategy. The recruitment of TAMs also faces significant challenges, including the compensatory accumulation of neutrophils that may exert immunosuppressive effects and the superfluous nature of the chemokine system, featuring numerous ligands and receptors exerting influence on monocytes. Circulating monocytes heavily rely on CCL2-CCR2 signaling to mobilize from the bone marrow and recruit to sites of inflammation.^112,331,332^ Thus, inhibiting CCR2 may forestall the mobilization of monocytes from the bone marrow, culminating in a diminution of the circulating cell reservoir and a reduction in the presence of TAMs at both primary and metastatic locales. In preclinical models, CCL2/CCR2 obstruction has been demonstrated to enhance the efficacy of chemotherapy, radiotherapy, and immunotherapy. However, the clinical effectiveness of this triple therapy (CCR2 inhibition, chemotherapy, and checkpoint blockade) requires further investigation.^333,334^ Additional pathways implicated in macrophage recruitment encompass the CXCL12/CXCR4 and angiopoietin-2/Tie2 axes. CXCL12 appears to play a role in recruiting Tie2+ macrophages, which are closely associated with blood vessels and contribute to tumor angiogenesis. Consequently, depleting Tie2+ macrophages can enhance vascular damage, and inhibiting Tie2 can prevent the recruitment of chemotherapy-induced Tie2+ TAMs, leading to reduced metastasis in breast cancer models.^335,336^ In another study, researchers developed ultrasmall copper nanoparticles (Cu@CuOx) targeted to CCR2 as nanocarriers loaded with gemcitabine. These nanoparticles precisely target CCR2 on monocytes/macrophages and effectively inhibit macrophage recruitment, thereby synergizing with the therapeutic effects of gemcitabine. This approach ultimately suppresses tumor progression and metastasis and extends the survival of patients with pancreatic ductal adenocarcinoma (PDAC) tumors treated with imaging-guided therapy.^337,338^

Another promising strategy is the induction of macrophage depletion, which effectively suppresses PMN-mediated immunosuppression and restores local immune surveillance. Bisphosphonates, which are absorbed by phagocytes, have cytotoxic effects on myeloid cells.^339^ Clodronate, a type of bisphosphonate, is swiftly recognized and phagocytized by macrophages, leading to apoptosis of host cells.^340^ Voluminous multi-micelle liposomes, encapsulating clodronate, have been meticulously engineered and adeptly employed across diverse cancer models, inhibiting tumor growth, angiogenesis, and metastasis.^341^ Additionally, trabectedin activates the Caspase 8 cascade through the TNF-related apoptosis-inducing ligand (TRAIL) receptor, which specifically targets mononuclear phagocytes, including macrophages. Reduced macrophage density has been associated with decreased angiogenesis in patients treated with trabectedin.^342^ CSF-1R inhibitors, such as BLZ-945, can also specifically target TAMs. Shen et al. developed pH-sensitive nanoparticles for the combined delivery of BLZ-945 and platinum to consume TAMs and kill tumor cells, achieving a combination of chemotherapy and immunotherapy.^343,344^ Studies have demonstrated the effectiveness of CSF-1R inhibitor-loaded nanoparticles in consuming TAMs and inhibiting tumor growth and metastasis.^344^ Surface markers on macrophages, like CD206, can likewise serve as therapeutic targets. Zang et al. developed a nano-therapeutic drug called lipid-coated calcium zoledronate nanoparticles (CaZol @ pMNPs) based on the concept mentioned above. These nanoparticles exhibited enhanced cell internalization and PEG detachment under low pH TME conditions. This indicated that mannose facilitated the delivery of calcium zoledronate to consume macrophages, reduced angiogenesis, and inhibited immunosuppression.^345^ However, it remains unclear whether macrophage clearance solely affects immunosuppressive myeloid cells or has broader implications. Therefore, further discussion is needed to determine the future prospects of this therapy.

While macrophage depletion may provide certain advantages in the initial stages of the disease, the main challenge lies in the difficulty of specifically targeting M2 macrophages using conventional drugs. Therefore, a more beneficial approach for eliminating tumors and inhibiting metastasis is to reprogram M2 macrophages, which promote tumor growth, into tumor-killing M1 macrophages, rather than completely removing macrophages. Yu et al. developed a magnetic Fe_3_O_4_ nanoparticle (MNPs@MDSCs) system by encapsulating MNPs within the MDSC membrane. This system, which easily aggregates in the TME, is capable of reprogramming M2-like macrophages into M1-like macrophages. Additionally, it induces immunogenic cell death, thereby enhancing anti-tumor activity.^346^ Moreover, the surface receptors exhibited by TAMs can attach to the Fc fragment of antibodies, enabling their participation in antibody-dependent cytotoxicity/phagocytosis (ADCC/ADCP). By targeting ADCP induced by the CD47-SIRPα axis, mouse macrophages in tumor models can be biased toward the M1-like phenotype, promoting the anti-tumor immune response.^347,348^ Zhang et al. developed nanogels assembled with anti-signal transducer and activator of transcription 3 (anti-STAT3) siRNA. These nanogels effectively antagonize M2 polarization, increase CD8 + T cell infiltration, block the STAT3 signaling pathway, trigger M1 macrophage activation, and ultimately exhibit significant efficacy in inhibiting tumor growth and metastasis.^349,350^ Toll-like receptors (TLRs) can also serve as targets for reprogramming macrophages. The TLR7 agonist Poly(I:C) has been shown to promote the enhanced anti-tumor function of TAMs. To exploit the codelivery characteristics of hydrophobic small molecule microbial TLR7 agonist and hydrophilic macromolecule anti-CD47 antibody, a nanoscale metal-organic framework was developed to repolarize macrophage M1 and activate the immune response. This strategy may facilitate the thorough elimination of both primary and distantly metastasized tumors in bilateral colorectal cancer models.^351–353^

In summary, reprogramming macrophages to exhibit an anti-tumor phenotype can not only hinder the growth of primary tumors but also weaken the immunosuppressive microenvironment formed by macrophages in PMN, preventing CTC colonization and thus inhibiting tumor metastasis. This approach represents a promising cancer immunotherapy.^354–356^ There are indeed drawbacks and limitations to targeted macrophage therapies in the PMN. First, the intricate functions and interactions of macrophages within the TME suggest that a singular targeted macrophage therapy may not comprehensively impede the metastatic process. Second, the heterogeneity and adaptability of macrophages pose challenges in devising precise targeting strategies, potentially resulting in fluctuating therapeutic effectiveness. Efforts to optimize targeted macrophage therapies must consider these complexities to enhance their efficacy in inhibiting metastasis. Furthermore, macrophages are pivotal for immune regulation and inflammatory responses, thus excessive manipulation of macrophages could detrimentally impact the body’s immune system. In conclusion, while targeted macrophage therapy holds promise in the PMN, thorough research and optimization are necessary to address its limitations and potential risks.

#### Neutrophils

Neutrophils are among the principle cells implicated in inflammation process and host defense against microbial infection.^357,358^ Their N2 polarization in the PMN not only induces immunosuppression, but also enhances the plasticity of CTCs promoting cancer cell colonization in the PMN. Therefore, the development of therapies targeting neutrophils is a viable approach for inhibiting cancer metastasis.

Inhibiting N2-polarized neutrophils in the PMN or polarizing them to N1 neutrophils is a viable treatment modality. Studies have shown that salidroside, a natural antioxidant, is effective at inhibiting neutrophil polarization towards the N2 phenotype within the PMN at lower concentrations. Additionally, it promotes the N1 phenotype by reducing the expression of activated STAT3.^164,359^ Similarly, blocking neutrophil recruitment in the PMN is also an effective measure. Activated neutrophil membrane-encapsulated nanoparticles (aNEM NPs) not only blocked neutrophil recruitment in the PMN, but also suppressed neutrophil adhesion to the tumor vascular endothelium and CTCs.^360^

Targeted NETs is also an effective inhibitor of PMN formation. NETs have been identified in the omentum of women with early-stage ovarian cancer and are able to bind ovarian cancer cells and promote omental metastasis. Neutrophil peptidylarginine deiminase 4 (PAD4) is critical for NET formation, and the use of pharmacological inhibitors of PAD4 can block NET formation and the colonization of cancer cells in the PMN.^361,362^ For example, the PAD4 isozyme-selective small molecule inhibitor JBI-589 reduces CXCR2 expression and prevents neutrophil chemotaxis, effectively inhibiting cancer metastasis.^363^ Recent studies have exploited the advantages of neutrophil chemotaxis and depletion of tumor-secreted factors to target PMN. Given the characteristic release of NETs from live neutrophils in the PMN that fosters tumor metastasis, live neutrophils are rapidly frozen as dead neutrophils (c NE), thus maintaining their role in inhibiting PMN formation. Subsequent formation of c NE^Mag^ by binding of the bacterial magnetosome to c NE was able to polarize M2 macrophages to M1 macrophages.^364^ Furthermore, CCDC25, functioning as a transmembrane protein on cancer cells, can interact with NETs, leading to cancer cell backbone rearrangement and directional migration. By preparing cell membrane hybridized liposomes stably expressing CCDC25, and encapsulating DNase I in the liposomes, we were able to effectively target and eliminate NET and significantly inhibit the recruitment of neutrophils, which could inhibit the PMN formation and cancer metastasis.^365,366^ Theoretically, the therapeutic strategy for targeting neutrophils is the same as that for targeting macrophages, and further clinical trials are necessary to ascertain the feasibility of implementing this approach in clinical settings.

#### Myeloid-derived suppressor cells

MDSCs, characterized as immature myeloid cells exerting immunosuppressive functions, serve as the source of a variety of immunosuppressive cells in the PMN.^5^ It is able to participate in multiple mechanisms of PMN formation and can remain present in the PMN after primary tumor resection. Therefore, the development of therapies targeting MDSCs could be of great benefit to cancer patients.^367,368^

The first measure is to inhibit the aggregation of MDSCs in the PMN. Researches have demonstrated that the chemokine CXCR2 participates in the recruitment of MDSCs. Therefore, SX-682, a CXCR2 antagonist, can effectively prevent the aggregation of MDSCs and enhance T cell activation.^369,370^ The CCL2-CCR2 chemokine pathway is also capable of inducing the directional migration of MDSCs into the PMN, and therefore the use of inhibitors of CCL2 or CCR2 is equally beneficial for minimizing the impact of MDSCs on the PMN.^371–373^ Epigenetic therapy, an emerging therapeutic approach, aims to treat disease by modulating epigenetic modifications of genes.^374^ Studies have shown that epigenetic therapies can also affect MDSCs in the PMN. The use of low-dose adjuvant epigenetic therapy (LD-AET) was able to inhibit cancer metastasis by blocking the aggregation of MDSCs in the PMN. And it was also able to antagonize cancer metastasis by converting MDSCs in the PMN to a mesenchymal macrophage phenotype.^317,375,376^ In addition, the traditional Chinese medicine Xiaoliu Pingyi recipe (XLPYR) was able to prevent the recruitment of MDSCs in the PMN, as well as reduce the expression of 100A8, S100A9, MMP9, and LOX, and down-regulate the IL-6/STAT3 pathway.^377^ Similarly, Chinese herbal medicine Baoyuan Jiedu decoction was able to inhibit MDSC aggregation in the PMN by suppressing the expression of genes and proteins such as TGF-β and Smad2 in the TGF-β/CCL9 signaling pathway, thereby inhibiting MDSC aggregation in the PMN.^378,379^ This proves that Chinese medicine may also be an effective way to inhibit tumor metastasis.^380^

The second measure is to remodel the immunosuppressive microenvironment induced by MDSCs.^381^ Prior research has indicated that tadalafil can downregulates the activities of iNOS and ARG1 to significantly inhibit the function of MDSCs, thus activating anti-tumor immunity.^382^ And good results have been achieved in clinical trials.^383,384^ STAT3 is a major transcription factor that produces immunosuppressive functions in MDSCs.^385^ Studies have shown that by combining STAT3 siRNA or decoy oligonucleotides with the TLR9 agonist CpG oligonucleotides, they are able to target TLR9-positive MDSCs and tumor cells, thereby reversing their immunosuppressive microenvironment.^386^ In addition, lipids accumulated in MDSCs are able to enhance immunosuppression by acting in a STAT3- and STAT5-dependent manner. Therefore, pharmacological suppression of fatty acid oxidation can suppress MDSCs-induced immunosuppression.^387,388^ Some scholars have reported that the construction of special nanomedicine delivery systems can also alleviate MDSCs-mediated immunosuppression and reduce the recruitment of MDSCs. This new nanosystem may be a major innovation for future therapeutic applications.^389–392^

The third approach is MDSCs depletion. It has been reported that the use of chemotherapeutic agents such as 5-fluorouracil (5FU), paclitaxel, cisplatin and gemcitabine not only depletes MDSCs, but also enhances anti-tumor activity in the PMN and inhibits the colonization of CTCs.^393–395^ In summary, multiple measures by targeting MDSCs may be an auspicious approach to impede the formation of PMNs.

#### T and B cells

The importance of treatments that target T and B cells within the PMN resides in thwarting tumor metastasis and improvement of therapeutic efficacy by preventing tumor cells from settling and growing in distant organs at an early stage through modulation of the immune environment and inhibition of the formation of the pre-metastatic microenvironment. This therapeutic strategy brings new hope and possibilities for tumor treatment.

The formation of the PMN is dependent on core immunosuppressive genes expressed by myeloid cells.^396^ Treatment of cancer metastasis by genetically engineered myeloid cells has become an emerging therapeutic strategy in recent years. IL-12 is able to stimulate the initiation and effector functions of T cells and NK cells, and induce a strong γ-interferon (IFNγ) response, thereby increasing anti-tumor functions.^397^ In vivo delivery of IL-12 by constructing genetically engineered myeloid cells (GEMys) not only activates antigen presentation and T cell activation, but also remodels the immunosuppressive microenvironment of the pre-metastatic ecological niche.^398^ The CD40 receptor, a member of the TNF receptor family, is expressed on antigen-presenting cells like macrophages.^399^ When it binds to its specific ligand, CD40L, found on the surface of activated T helper lymphocytes, the CD40 receptor induces the production of TNF, ROS, and various other factors. These molecules play a crucial role in the bactericidal and tumoricidal activities of macrophages. In preclinical studies, the administration of CD40 agonist monoclonal antibodies (mAbs) has successfully converted immunosuppressive TAMs into cytotoxic effectors, leading to enhanced immune surveillance and the inhibition of tumor cell colonization and growth in the metastatic niche.^330^ CD47 is presented on normal cells and serve as a “don’t eat me” signal, indicating that SIRPα-expressing mononuclear phagocytes and neutrophils are protected from host cell removal.^400,401^ Activation of the CD47-SIRPα signaling pathway allows tumor cells to evade phagocytosis by macrophages, thereby maintaining survival and invasive capacity in PMN. Targeting the CD47-SIRPα axis can enhance the phagocytic capacity of macrophages, thereby augmenting their antigen burden and amplifying the presentation of antigens to T cells.^402^ This approach has the advantage of simultaneously boosting the immune system’s capacity to identify and eradicate tumor cells.

However, for PMN, therapies targeting T and B cells, which are adaptive immune cells, are induced by TDSFs, MDSCs, and EVs to promote the formation of PMN and the colonization of CTCs, have some drawbacks. Such a therapy is clearly flawed, as metastasis inhibition requires primary factors, and therapies targeting T and B cells can only assist in promoting anti-tumor function. Therefore, therapeutic strategies targeting B and T cells must be integrated with additional therapeutic approaches to optimize benefits.

### Targeting molecular components in pre-metastatic niche formation

#### Tumor-derived secretory factors

Therapies targeting TDSFs are important in the PMN. This therapeutic approach not only intervenes in the initial phases of tumor cell metastasis but also prevents the establishment of the pre-metastatic microenvironment, but also affect the immunosuppression and angiogenesis of the TME through mechanisms such as regulating the immune environment and inhibiting angiogenesis, thus preventing distant metastasis. By targeting TDSFs, the invasion and proliferation of tumor cells can be effectively blocked, and the therapeutic effect and survival rate can be improved, bringing new hope and breakthroughs in tumor treatment.

TDSFs are involved in numerous steps and mechanisms underlying PMN formation. TDSFs, including G-CSF, VEGF-A, PLGF, TGF-β, S100 protein, and TNF, play crucial roles in PMN formation.^24^ Targeting TAMs by inhibiting the CSF-1R signaling pathway, a crucial macrophage signaling pathway, has been s demonstrated to diminish tumor growth and metastasis. Interestingly, blocking CSF-1R or CSF-1 signaling has been found to increase metastasis in breast tumors (such as 4T1.2 and EMT6.5), and patients with disease progression may exhibit a better response to G-CSFR therapy.^403^ Recent studies have revealed that scutellarin inhibits RUNX1 activation and reduces G-CSF production in triple-negative breast cancer-associated endothelial cells by targeting TNF-α/TNFR2, thereby suppressing triple-negative breast cancer metastasis.^404^ In vitro and in vivo experiments have demonstrated that histidine-rich glycoprotein can decrease PLGF expression, leading to a shift in TAM polarization from an M2 to an M1-like phenotype, thereby inhibiting tumor growth, metastasis, and vascular abnormalities.^405^ Similarly, S100A8 promotes the migration of CRC cells in the inflammatory microenvironment by activating the NF-κB pathway and inducing the overexpression of miR-155, IL-1β, and TNF-α. Targeting S100 protein represents a viable strategy to prevent PMN formation.^406^ In conclusion, targeting specific molecules or cells involved in macrophage interactions can effectively inhibit the mobilization and recruitment of BMDCs, as well as prevent the colonization of myeloid cells in the niche prior to distant organ metastasis in the future. However, TDSFs are less specific and it is difficult to distinguish whether they are of tumor cell or normal cell origin. Consequently, further research is imperative to meticulously assess the precision and effectiveness of this therapeutic strategy.

#### Extracellular vesicles

EVs derived from tumors critically facilitate the transfer of genetic information between tumor cells and basal cells, fostering angiogenesis and enhancing tumor growth and invasion. The formation of PMN is dependent on tumor-derived EVs.^407,408^ Therefore, intercepting the delivery of EVs carriers to recipient cells presents a promising strategy for preventing tumor metastasis.

Reducing EV induction in the PMN by targeting proteins involved in EV production and secretion from tumor sources may be an effective therapeutic modality.^409,410^ In a variety of cancers, cannabidiol use is effective in reducing the release of cancer-derived exosomes in a dose-dependent manner. The mechanism of action may involve inhibiting the release of EVs by modulating mitochondrial metabolism.^411^ Calpain promotes apoptosis, cell proliferation, migration, tumor invasion, release of EVs and cancer progression in cancer. The use of calpeptin, a calpain inhibitor, inhibits shedding of EVs and reduces the release of EVs.^412,413^ Rho-associated protein kinase (ROCK) regulates cell shape and motility by acting on the cytoskeleton, a process that promotes shedding and release of EVs. Y27632, an inhibitor of ROCK1 and ROCK2, blocks proteins involved in cell motility, thereby reducing the release of EVs.^414,415^ These studies demonstrated that cancer progression can be effectively inhibited by inhibiting the release of EVs. However, more time is needed to explore how to selectively release those EVs involved in normal physiological processes and block the release of EVs involved in pathological processes.^416^

Exosomes are able to function as vehicles for drug delivery owing to their inherent molecular transport capabilities, good biocompatibility, long circulation time, and ability to penetrate deep tissues. A past phase I clinical trial in patients with stage III/IV melanoma used DCs-derived exosomes carrying functional MHC/peptide complexes with favorable results.^417^ The ability of exosomes to encapsulate adeno-associated viruses to transfer genes extensively to the retina was shown to be greater than that of adeno-associated viruses alone.^418^ However, exosomes derived from immune cells in vivo are only capable of serving as drug delivery vehicles and do not produce sufficient effects to inhibit tumor progression.^419^ Engineered exosome therapy was also developed. Engineered exosomes were obtained by mounting conjugate-modified CD47 and SIRPα antibodies to azide-modified exosomes. Exosomes, when administered in vivo, was able to block SIRPα and CD47 on macrophages, respectively, and improve the phagocytosis of macrophages. It was also able to achieve macrophage reprogramming and inhibit cancer metastasis.^420^ Mutations in GTPase KRAS can promote cancer progression and metastasis in pancreatic cancer. By designing engineered exosomes carrying siRNA or shRNA targeting the oncogene KRAS^G12D^, cancer and metastatic progression can be effectively inhibited and survival can be improved.^421,422^ This finding demonstrated the feasibility of exosomes as a drug delivery modality.

Many studies have proved that the secretion of exosomes from cancer cells can promote the proliferation, invasion and metastasis of tumor cells, thus promoting the development and metastasis of cancer.^423,424^ Therefore, exosomes may also serve as biomarkers for facilitating early diagnosis, disease monitoring and prognosis assessment of tumors.^425^ As small vesicles that carry abundant biological information, exosomes play a pivotal role in liquid biopsy. By analysing biomarkers in exosomes, early diagnosis, disease monitoring and prognosis assessment of tumors can be achieved, predicting treatment response and guiding the selection of personalized treatment plans. Exosomes can also reflect tumor immune status and monitor the effect of immunotherapy.^426–429^ The application of exosomes in liquid biopsy brings new ideas to the field of medical oncology, which is expected to achieve more accurate tumor treatment and monitoring and improve the quality of patient survival.^430^

### Targeting circulating tumor cells

CTCs that are released from from primary tumors into the circulatory system via blood or lymphatic vessels. They enhance tumor growth and metastasis in target organs through invasion, migration, and complex intercellular signaling interactions.^431,432^ It has been discovered that NK cells can directly or indirectly engage with CTCs to regulate cancer metastasis. CTCs undergoing EMT lose inhibitory ligands that inhibit NK cells, leading to the ability of NK cells to kill CTCs.^433^ Therefore, driving NK cell activity in vivo is a viable approach for targeting CTCs. Targeted NK cell activation, interleukin therapy, and adoptive NK cell therapy have shown promising applications.^434–436^ However, more clinical trials are needed to demonstrate the effectiveness of these therapies and their potential for clinical translation.

Circulating tumor DNA (ctDNA) is a free DNA fragment released by tumor cells into the bloodstream that carries the genetic information of the tumor cells.^437,438^ Both can be detected and analysed by liquid biopsy methods, thus providing comprehensive information and guidance for tumor diagnosis, treatment and monitoring. Liquid biopsy is a non-invasive biospecimen collection method used to analyse the onset, progression, and prognosis of a disease by collecting biomarkers such as cellular debris, DNA, and RNA from a patient’s body fluids.^439,440^ Before distant metastases can form, CTCs need to migrate into PMN suitable for colonization and growth. The risk of metastasis in a patient can be assessed by testing the number and characterization of CTCs in the blood of the tested patient.^441^ A high level of CTC count represents the ability of the primary tumor to infiltrate into the vasculature on the one hand, and the likelihood of metastasis formation in distant organs on the other. Liquid biopsies of CTCs play a role in detection, prognosis and progression testing in a wide range of cancer tests.^425,441–444^ Currently, several techniques can be applied to isolate CTCs: immunomagnetic bead sorting, microfluidic sorting and enrichment system, density gradient centrifugal enrichment, etc.^445,446^ The CellSearch system is the first FDA-approved method for CTC detection. The CellSearch system utilizes EpCAM to bind to magnetic beads with specific antibodies, employing the principle of shunting under the influence of an external magnetic field to effectively separate and purify CTCs, offering dependable tools and technical assistance for the diagnosis and treatment of cancer. However, this technology is currently expensive to use, applicable only to specific types of cancer, and cannot realize real-time monitoring. Therefore, its widespread clinical application requires the support of clinical trials.^447,448^

Targeted ctDNA plays a pivotal role in the PMN. Quantitative and qualitative analysis of pre-metastatic ctDNA can be achieved by molecular labeling methods, next-generation sequencing, digital PCR and high-sensitivity mass spectrometry. These methods can not only help diagnose tumors at an early stage, but also predict the risk of metastasis, monitor treatment efficacy and predict prognosis.^449,450^ The analysis of pre-metastatic ctDNA helps to individualize the treatment plan and improve the treatment effect and survival rate. In addition, by monitoring pre-metastatic ctDNA changes, it can reflect tumor progression in real time and guide the adjustment of treatment plans. Early detection and intervention in the pre-metastatic TME can effectively prevent tumor metastasis and recurrence, and improve patient quality of life and survival rate. However, ctDNA only accounts for a small portion of cell-free DNA (cfDNA), from which it is very challenging to precisely isolate ctDNA.^451^ Only the detection of tumor-specific mutations (gene copy number variation, methylation modification, single nucleotide mutation) in cfDNA can indicate the presence of ctDNA.^452–454^ Therefore, the presence of targeted ctDNA and CTC in PMN is important for early diagnosis, treatment and monitoring of tumors, providing patients with more individualized treatment options and care.

### Signaling pathways targeting pre-metastatic niche formation

#### TGF-β signaling pathway

The TGF-β signaling pathway is a pivotal cellular signaling pathway that governs diverse cytological processes such as immunosuppression, growth inhibition, cell migration, invasion, and ECM remodeling.^455,456^ The main mechanism is as follows: TGF-β molecules bind to TGF-β receptor II on the cell membrane, which activates and phosphorylates it, which in turn activates TGF-β receptor I. Activated TGF-β receptor I phosphorylates Smad proteins, which induces the formation of a complex between Smad2/3 and the cotranscription factor Smad4, which penetrates the nucleus and orchestrates the transcription of target genes, thereby influencing the cellular physiological functions.^457^ During the initial phase of tumor development, TGF-β frequently functions as a tumor suppressor, preventing early tumors from progressing to malignancy. In later stages, TGF-β is often expressed in high abundance in tumor tissues, and the signaling pathway is activated abnormally for a long period of time, which counteracts its anti-proliferative effect and changes its function to promote tumor metastasis. The TGF-β signaling pathway is associated with many metastatic processes and significantly affects the ability of tumor cells to spread throughout the body.^457–459^

Increasing clinical evidence has revealed that aberrant activation of the TGF-β signaling pathway can also facilitate tumor metastasis to specific tissues and organs by selectively up-regulating the expression of organ-specific metastasis-related genes.^460–462^ Therefore, targeting the TGF-β signaling pathway can impede the establishment of PMN of tumor cells and reduce the risk of tumor metastasis, thereby enhancing patient survival rates. Currently there are three main ways to target the TGF-β signaling pathway: a. Inhibitors of TGF-β secretion (antisense oligonucleotides).^463^ b. Blocking of TGFβ and its interaction with the receptor by monoclonal antibodies or soluble TGF-β receptor, such as anti-TGFβ antibodies, anti-receptor antibodies, TGF-β trap receptor ectodomain proteins, and small-molecule inhibitors targeting the TGF-β receptor kinase. c. Inhibition of the TGF-β signaling pathway by kinase inhibitors or aptamers interferes with the function of downstream Smad signaling proteins. New therapies that target TGF-β production or block its action are currently in preclinical or early clinical trials and have shown promise.^464–466^

#### VEGF signaling pathway

The VEGF signaling pathway is a crucial angiogenic signaling pathway primarily engaged in the regulation of vascular endothelial cell proliferation, migration, and angiogenesis.^467,468^ The VEGF signaling pathway includes a variety of VEGF family members (e.g., VEGF-A, VEGF-B, VEGF-C, VEGF-D, etc.) as well as their receptors (VEGFR-1, VEGFR-2, VEGFR-3) and signaling molecules (e.g. PI3K-Akt, Ras-MAPK, etc.).^469^ In the PMN VEGF provides sufficient nutrients and oxygen to tumor cells by promoting angiogenesis and increasing vascular permeability, as well as providing a pathway for tumor cells to invade and metastasize. Therefore, therapies targeting the VEGF signaling pathway have a wide range of applications for inhibiting PMN formation.^470^

The treatment methods targeting VEGF signaling pathway mainly include anti-VEGF drugs and VEGF receptor inhibitors.^471,472^ These drugs can inhibit the activation of the VEGF signaling pathway through different mechanisms, block angiogenesis and reduce vascular permeability, thereby inhibiting tumor growth, metastasis and the formation of PMN. Anti-VEGF drugs mainly include bevacizumab and sunitinib. They inhibit the binding of VEGF-A to VEGFR and block VEGF signal transduction, thereby inhibiting tumor angiogenesis and growth. VEGF receptor inhibitors such as sorafenib and ramucirumab inhibit tumor growth and angiogenesis by inhibiting the activation of VEGFR and blocking its signal transduction.

#### MET signaling pathway

MET signaling pathway is an important receptor tyrosine kinase signaling pathway, whose receptor is Mesenchymal Epithelial Transition Factor (MET), also referred to as the Hepatocyte Growth Factor Receptor (HGFR).^473^ The main ligand of the MET signaling pathway is Hepatocyte Growth Factor (HGF), and when HGF binds to the MET receptor, it activates the tyrosine kinase activity of the MET receptor, which initiates a cascade of downstream signaling pathways., including PI3K/AKT, MAPK/ERK, and so on.^474,475^

Activation of the MET signaling pathway in the PMN not only enhances tumor cell migration and invasion, but also promotes neo-angiogenesis.^476^ MET shows aberrant activation in a variety of cancers, which is intimately associated with the growth, proliferation and invasive ability of tumor cells.^477^ Therefore, blocking the HGF/MET signaling pathway can effectively inhibit tumor development and metastasis. Currently, there are three main types of HGF/MET inhibitors: biological antagonists, monoclonal antibodies, and small molecule inhibitors.^478^ Biological antagonists include proteins or peptides that inhibit the binding of HGF to MET, thereby blocking the HGF/MET signaling pathway. Biological antagonists that act on the HGF/MET signaling pathway mainly include HGF variants NK1, NK2 and NK4. Their mechanism of action is to compete with HGF ligands for binding to MET and inhibit the tyrosine phosphorylation of MET receptors induced by HGF, thus reducing the activity of the HGF/MET pathway. Monoclonal antibodies inhibit the interaction by targeting either the MET receptor or HGF, thereby inhibiting the activation of the signaling pathway. Small molecule inhibitors (Tivantinib and INC280), on the other hand, inhibit signaling by interfering with the tyrosine kinase activity of the MET receptor.^479–481^ Targeting the HGF/MET pathway with biological antagonists, monoclonal antibodies, and small molecule inhibitors offers promising therapeutic avenues for inhibiting tumor growth and metastasis. By disrupting the aberrant activation of MET in various cancers, these inhibitors hold potential for improving patient outcomes and advancing the treatment of metastatic disease.

## Conclusion and future perspectives

In this review, we have delved into a crucial concept in tumor metastasis - the PMN, which provides the fertile ground for the distant spread of cancer. By studying the cellular and molecular constituents of the PMN, we have revealed its formation mechanisms and discussed the landscape of the PMN in different metastatic organs. Furthermore, we have also discussed therapeutic approaches targeting the PMN, including targeted therapies against its cellular and molecular components, as well as interventions targeting CTCs and signaling pathways. Importantly, therapies targeting the PMN may have different effects on different metastatic organs. This is because the microenvironment and cellular composition of different organs may affect the growth and metastasis of tumor cells.^482^ For example, organs such as the lung and bone have different cell types and growth factors and may require different interventions to prevent tumor cells from metastasizing.

**Fig. 6:**
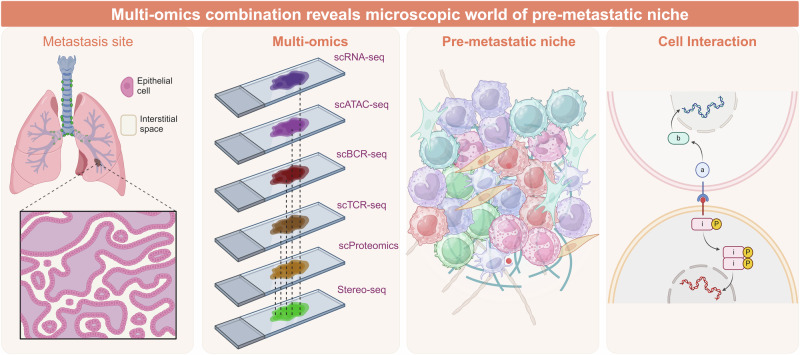
Applications of single-cell omics, proteomics, and spatial transcriptomics in pre-metastatic niche analysis: Envisioning the future. Highlights the potential applications of single-cell omics, proteomics, and spatial transcriptomics in pre-metastatic niche analysis, offering a glimpse into the future of research in this area. These cutting-edge technologies enable researchers to delve deeper into the molecular and cellular landscape of the pre-metastatic niche, providing a more comprehensive understanding of the mechanisms underlying tumor metastasis

Research on the PMN is essential for a better understanding of the tumor metastasis process. By probing deeper into the mechanisms governing its formation, we can identify new therapeutic targets, providing crucial insights for devising more efficacious strategies to prevent and treat tumor metastasis. In this research field, new insights and discoveries continue to emerge, revealing the complexity and diversity of tumor metastasis. In future research, we can further explore the association between the PMN and tumor metastasis, investigate the mechanisms of action of different cellular and molecular components in the PMN, and develop more targeted treatment strategies. Additionally, by integrating the latest technological tools such as single-cell transcriptomics, proteomics and spatial transcriptomics, we can comprehensively unravel the complex network of the PMN, providing more precise guidance for personalized medicine (Fig. 6).^483^

In conclusion, we provided a comprehensive and in-depth exploration of the critical role of the PMN in tumor metastasis, laying the foundation for further research into the mechanisms of tumor metastasis and the development of new treatment strategies. We hope that our efforts can provide new insights for future tumor metastasis research and clinical practice, contributing to the fight against this formidable challenge. Through the study of the PMN, we can better understand the complexity and diversity of tumor metastasis, offering new perspectives for the development of more effective treatment strategies. The PMN, as a key link in tumor metastasis, will continue to be a hot topic in cancer research. However, there are still several problems to be solved, including (1) Can CTCs continue to survive after surgical resection of the primary tumor? Can PMN continue to form? Currently, some patients in the clinic still recur or form tumors in other areas after the primary tumor is excised. (2) Can the immune system inhibition caused by chemotherapy and radiotherapy after surgery provide an opportunity for the establishment of PMN? Postoperative radiotherapy remains the mainstay of treatment for patients with tumor resection in several countries. (3) Can the therapy targeting PMN play a corresponding role? (4) Do all primary tumors form the PMN? It is not yet definitively established whether all primary tumors give rise to the PMN, as this phenomenon may vary depending on tumor type and individual factors. These above issues still need further robust research to prove and reveal them. In this ever-evolving field, continuous exploration, innovation, and collaborative efforts are needed to achieve a deep understanding of tumor metastasis and effective interventions.
